# The role of autoantibodies in Alzheimer's disease: Pathogenetic connections or epiphenomena?

**DOI:** 10.1002/alz.70484

**Published:** 2025-07-22

**Authors:** Miyo K. Chatanaka, Ioannis Prassas, Colin L. Masters, Eleftherios P. Diamandis

**Affiliations:** ^1^ Department of Laboratory Medicine and Pathobiology University of Toronto Toronto Ontario Canada; ^2^ Laboratory Medicine Program University Health Network Toronto Ontario Canada; ^3^ The Florey Institute of Neuroscience and Mental Health University of Melbourne Melbourne Victoria Australia; ^4^ Lunenfeld‐Tanenbaum Research Institute Sinai Health System Toronto Ontario Canada

**Keywords:** Alzheimer's disease, Alzheimer's disease diagnosis, Alzheimer's disease therapy, autoantibodies, autoimmunity, cerebrospinal fluid, early diagnosis, mass spectrometry, neurodegeneration, neuroinflammation

## Abstract

**INTRODUCTION:**

The current evidence supporting the complex, multifaceted etiology for Alzheimer's disease (AD) grows by the day, prompting increased research in non‐“amyloid hypothesis”‐related pathways. One of these pathways of interest pertains to an autoimmune component in this disease.

**METHODS:**

In this review, we briefly discuss current evidence of potential contributions of autoimmunity to AD pathobiology and describe the putative role of autoantibodies detected in patient fluids. We draw attention to the fact that the reported AD‐related autoantibodies differ dramatically between published studies, raising doubts about the reliability and robustness of these findings.

**RESULTS:**

We hypothesize, and provide indirect evidence, that many of the reported autoantibodies in AD may represent false discoveries. We suggest follow‐up validation and confirmatory studies with sufficient power, preferably by employing orthogonal testing techniques.

**DISCUSSION:**

Uncovering the putative autoimmune components of AD is important and could pave the way to new concepts for AD pathogenesis, diagnosis, and therapy.

**Highlights:**

- Although Alzheimer's disease (AD) is not traditionally considered an autoimmune disease, growing evidence suggests immune system dysregulation and autoantibody generation, either in the form of naturally occurring or pathogenic autoantibodies.
- Numerous studies have discovered autoantibodies in AD, but only a few of them have been found independently and multiple times, including amyloid β (Aβ) and tau autoantibodies.
- Many of these findings represent false discoveries.
- Follow‐up validation and confirmatory studies with sufficient power are imperative, preferably by employing orthogonal testing techniques.
- Understanding the immune and autoimmune landscape in AD will assist in future immunotherapy strategies.

## 1 BACKGROUND

### 1.1 Autoimmune diseases

Autoimmune diseases manifest clinically when the immune system mistakenly attacks its own healthy cells and bodily tissues, leading to a wide range of multi‐organ symptoms.^1^ In the general population, autoimmune diseases are very common, with an estimated prevalence of 10%, which increases with age.^2^ A number of autoimmune diseases are pathogenetically linked with the presence of one or more “signature” autoantibodies targeting normal self‐tissues. These autoantibodies frequently serve as diagnostic and prognostic markers for these diseases. Some examples of common autoimmune diseases and their associated pathogenetic/diagnostic autoantibodies are shown in Table 1.

**TABLE 1:** Common autoimmune diseases and their pathogenetic/diagnostic autoantibodies detected in serum or tissues.

Autoimmune disease	Associated pathogenetic/diagnostic antibody
Rheumatoid arthritis	Rheumatoid factor (RF), anti‐citrullinated protein antibodies
Systemic lupus erythematosus	Antinuclear antibodies (ANA), anti‐double‐stranded DNA (dsDNA) antibodies, anti‐Smith antibodies (Sm)
Hashimoto's thyroiditis	Thyroid peroxidase antibodies (TPOAb), thyroglobulin antibodies (TgAb)
Type 1 diabetes	Islet cell antibodies (ICA), anti‐insulin antibodies, glutamic acid decarboxylase antibodies

The spectrum of autoimmune conditions with distinct clinical features and unique autoantibody profiles is continuously expanding. A detailed description of these disorders is beyond the scope of this review. It is important to mention that a number of common and rare autoimmune disorders are due to a multitude of pathogenetic mechanisms, in addition to autoimmunity.

#### 1.1.1 Common methods for autoantibody identification

Autoantibody identification involves various methods to detect antibodies produced by the immune system against self‐antigens. Nearly all methods are based on the formation of an autoantibody‐cognate antigen complex, followed by detection of the immunocomplex with anti‐human labeled antibodies.^1^ A brief summary of each method is mentioned in Table 2. It is important to mention which autoantibody method is used in literature reports, because different methods may generate different results.

**TABLE 2:** Various methods for autoantibody identification with a brief description.

Identification method	Description
Immunofluorescence (IF)	This method uses fluorescently labeled detection antibodies to detect autoantibodies bound to tissue or cell substrates. The pattern and intensity of fluorescence provide information about the type and specificity of autoantibodies present.
Enzyme‐linked immunosorbent assay (ELISA)	ELISA detects autoantibodies by using enzyme‐conjugated secondary antibodies that produce a color change when they bind to the antigen‐autoantibody complex. ELISA is highly sensitive, convenient, and economical, and is widely used for screening and quantitative analysis of autoantibodies.
Cell‐based assay (CBA)	CBAs utilize cultured cells expressing specific antigens to detect autoantibodies present in patient serum. The binding of autoantibodies to the cells is visualized using fluorescence or other detection methods, providing information on cellular localization and antigen specificity.
Western bot	This technique separates proteins based on size using gel electrophoresis. The proteins are then transferred to a membrane. Autoantibodies are detected by incubating the membrane with patient serum/fluids and then detecting autoantibody‐antigen complexes using labeled secondary antibodies.
Protein microarrays	Protein microarrays consist of immobilized proteins or peptides representing various antigens. Patient serum is incubated with the microarray, and autoantibodies binding to specific antigens are detected using fluorescent or chemiluminescent labeling. The immobilized proteins can be synthesized in vitro or expressed by phage libraries.
Immunoprecipitation (IP)	IP isolates autoantibody‐antigen complexes from complex biological samples using specific antibodies immobilized on a solid support. The complexes are then eluted and analyzed by techniques such as mass spectrometry or immunoblotting to identify the antigens.
Immunohistochemistry (IHC)	IHC detects autoantibodies in tissue sections using labeled antibodies that bind to the autoantibodies that have bound to specific antigens. It's commonly used in histopathology.
Line immunoassays	These assays utilize strips or membranes with immobilized antigens, allowing the detection of multiple autoantibodies simultaneously in a single test. They are useful for autoimmune disease diagnosis and monitoring.
Luminex assays	These assays employ microspheres with distinct fluorescent signatures, each coated with a different antigen. Patient serum is incubated with the microspheres, and autoantibodies binding to specific antigens are detected using fluorescently labeled secondary antibodies and flow cytometry.
Bead‐based assays	These assays utilize protein G‐coated magnetic beads that bind to autoantibodies of interest. Externally added antigens can be used to bind to the autoantibodies, and the complex can be identified through mass spectrometry or another protein method.
Multiplex Immunoassays	These assays allow for the simultaneous detection of multiple autoantibodies within a single sample using protein microarrays or bead‐based platforms. They offer high‐throughput screening and are valuable for identifying autoantibody profiles associated with specific diseases.

High throughput methods such as protein or phage microarrays have historically been utilized for novel autoantibody marker discovery, but they are accompanied by caveats such as cost, library availability and optimization requirements, and high background signals.^2^ Mass spectrometry presents an attractive and relatively new alternative to the methods described above because it enables proteome‐wide coverage, simultaneously testing the whole autoantibody reactome without requiring well‐characterized and purified antigens for assay development. Unfortunately, mass spectrometry suffers from poor reproducibility and, at times, low specificity.^3^

#### 1.1.2 Autoantibody assay limitations

It is highly important to recognize that all these autoantibody detection methods have assay‐specific limitations, which can lead to erroneous findings, such as false positives. Some reasons for false‐positive results with these methods are listed in Table 3.

**TABLE 3:** Some possible reasons for false autoantibody identification.

Clinically related reasons	Analytical method reasons
Heterogeneous patient populations with variable disease severity Differences in gender, ethnicity, race, age, and other demographic indices Presence of more than one autoimmune disease Testing after therapy initiation and the nature of drugs used	Most autoantibody detection methods are qualitative or at best, semiquantitative Standard preparations and quality control samples are not usually available The sensitivity of most assays is limited, which could lead to false‐negative results Large analytical variability (poor assay precision) Assay specificity (cross‐reactivity of autoantibodies with unrelated antigens) Heterophilic antibodies* and other interferents Most published results are not usually verified with orthogonal techniques Variable and arbitrary definition of sample positivity or negativity, making comparison between methods problematic Different methods used in different studies Limited proteomic coverage Epitope recognition by autoantibodies may vary considerably between patients

* Heterophilic antibodies are antibodies produced against poorly defined antigens. These are generally weak antibodies with multi‐specific activities. Human anti‐animal antibodies that develop because of treatments with animal immunoglobulins are antibodies with strong avidities, produced against well‐defined antigens.

In summary, most of the autoantibody detection methods are qualitative or semiquantitative at best, and standard preparations and quality control samples are not usually available. The lack of standards precludes the construction of calibration curves and quantification. This makes the comparison of findings between investigators difficult. The lack of unequivocal positive and negative quality control samples forces investigators to use patient specimens, which, at times, may not be immediately available in unlimited quantities. The sensitivity of these assays is also limited, which could lead to false‐negative results. The most widely used methods, based on the enzyme‐linked immunosorbent assay (ELISA) principle, may demonstrate significant differences in sensitivity, if the ELISA was developed in house or if it was purchased from commercial sources. These and other limitations affect the patient sample classification as being autoantibody‐positive or negative. The analytical variability is one major factor of disagreement between published results regarding positivity or negativity for a given autoantibody (please see later in this review). The majority of these techniques do not cover the whole proteome.^4^ For these reasons, any positive samples for certain autoantibodies, whenever possible, should be verified with orthogonal techniques that are based on different analytical principles. Newer autoantibody detection techniques are more sensitive and highly multiplexed, such as antigen arrays and mass spectrometry.^5^, ^6^, ^7^ When interpreting such data, it is difficult to select an unbiased cutoff for positivity or negativity, mainly because the assay background signals can vary widely between methods and between samples. One important caveat is that almost all of the above techniques are vulnerable to the presence of heterophilic antibodies, which are able to link the putative autoantibody and the labeled detection anti‐human antibody without the presence of the antigen.^8^ This phenomenon generates false‐positive data and has already been described in detail in the literature.^8^ We suggest that newly discovered putative autoantibodies are verified extensively by using positive identification techniques like mass spectrometry and by excluding the presence of heterophilic antibodies in the positive samples. ELISA kits that detect heterophilic antibodies in specimens are commercially available by many vendors.

### 1.2 Alzheimer's disease

#### 1.2.1 Amyloid hypothesis

The classic “amyloid hypothesis” for Alzheimer's disease (AD) has been previously described in detail.^9^ Briefly, it posits that amyloid precursor protein is processed by the enzymes β‐secretase and γ‐secretase to release amyloid β (Aβ) fragments, which aggregate into toxic oligomers, resulting in the formation of mature fibrils and extracellular Aβ plaques. During this process, there is also the formation of twisted fibers of phosphorylated tau proteins inside neurons. Cholinergic neuron damage, neuroinflammation, and oxidative stress are also important mediators of the disease.^10^ Together, these mediators and processes induce neuronal cell death and synaptic loss in AD.

Inflammation is thought to be integral in the pathogenesis of AD. Neutrophils, which are the first responders of the innate immune system and inflammation^11^ have readily been documented in the disease, particularly near Aβ plaques and in the periphery.^12^, ^13^, ^14^ Notably, certain neutrophil phenotypes have been associated with the rate of cognitive decline in AD,^13^ and an elevated neutrophil‐to‐lymphocyte ratio, which is an indicator of peripheral inflammation, has been seen in AD and co‐occurring conditions, including diabetes and hypertension.^15^, ^16^, ^17^ Another component of the innate immune system seen in AD is the nucleotide‐binding domain leucine‐rich repeat and pyrin domain containing receptor protein 3 (NLRP3) inflammasome,^18^ which is transcribed by an activated NF‐κB.^19^ Activation of the NLRP3 inflammasome inside microglia regulates the proinflammatory cytokine interleukin (IL) ‐1β, inducing neurotoxicity via glutamate production,^20^ drives tau pathology, and disrupts Aβ phagocytosis by microglia.^21^, ^22^ Finally, microglia, which are indisputably taking part in the pathobiology of AD, can be activated by Aβ plaques bound to complement component C1q and release pro‐inflammatory cytokines such as IL‐6 and tumor necrosis factor‐α (TNF‐α).^23^

It is now clear that Aβ and tau protein aggregation present an incomplete picture of the complex AD pathology. By the age of 80–85 years, Aβ aggregates are present in up to 40% of cognitively healthy elderly persons,^24^, ^25^, ^26^ and despite extensive trials in patients with AD, therapeutic targeting of processes and intermediates of the amyloid hypothesis has only marginally decreased the speed of disease progression, rather than treat it. Not surprisingly, immune pathways have consistently emerged as a contributor to AD in genome‐wide association studies (GWAS), although they have not been explored as extensively as traditional amyloid pathologies. Below, we provide published evidence that AD pathogenesis may, at least in part, be driven by aberrant autoimmune reactions, whereby host antibodies (autoantibodies) target self‐proteins in the brain and elsewhere, promoting neuroinflammation to an extent, followed by neuronal death. We also describe the use of methods for autoantibody identification and relate it to the current deficiencies of these methods (Table 3). Understanding the limitations of these assays will prevent or reduce false discoveries, defined as literature‐reported autoantibodies, which likely represent artifacts of the detection method or other factors instead of participating in disease pathobiology.

#### 1.2.2 Autoimmunity and AD

**FIGURE 1:**
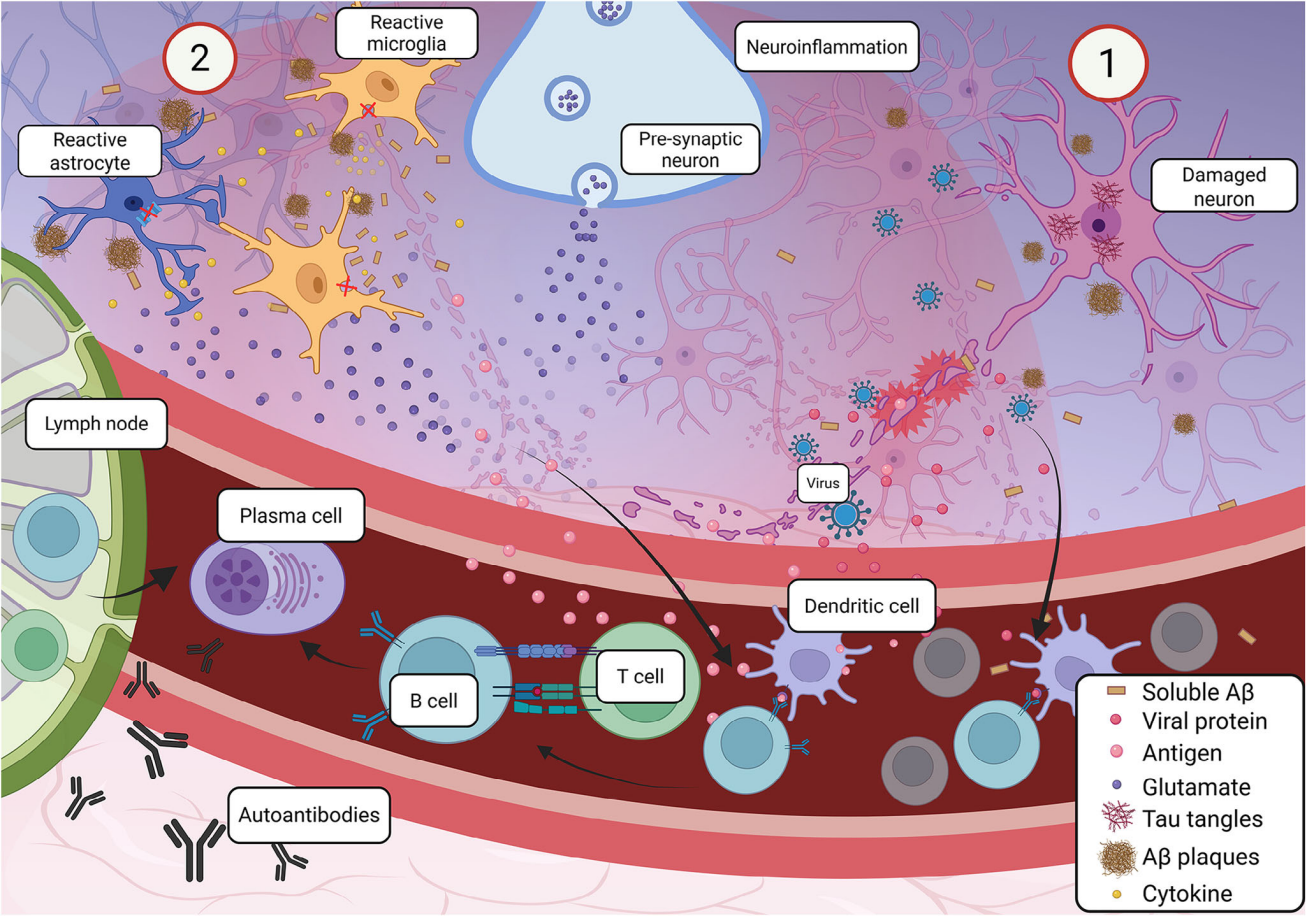
Schematic of proposed autoantibody generation in AD. Infections (1) and/or increased soluble Aβ (2) in the brain and the CSF can have downstream effects that lead to immune activation and antibody generation. In case (1), infectious agents (viruses, bacteria) can damage neurons, which in turn can release antigens into the circulation. These antigens can be recognized by dendritic cells or non‐deleted autoreactive B‐cells, thus creating a cascading effect with the help of autoreactive T‐cells and leading to antibody generation against neuronal/brain proteins. In addition, viral proteins can induce autoreactive B‐cells through molecular mimicry. *This hypothesis is showcased as part of a possible autoantibody‐generating immune mechanism*. Alternatively (or in parallel), in case (2), the increased production of Aβ fragments leads to microglial activation and eventual exhaustion. This damages neurons. Microglia become inefficient at clearing Αβ fragments. Simultaneously, astrocytes become reactive due to the inflammatory factors secreted by microglia (among other reasons, such as transforming growth factor‐α, interleukin‐1α, and tumor necrosis factor‐α. This leads to increased secretion of Aβ due to these reactive astrocytes, as well as inflammatory molecules that exacerbate neuroinflammation and neuronal dystrophy. Reactive astrocytes have several molecular alterations, with increased expression of genes such as glial fibrillary acidic protein (GFAP), S100 calcium‐binding protein B (S100B), TIMP metallopeptidase inhibitor 1 (TIMP1), chemokines, and complement proteins. Astrocytes also lose their glutamate uptake abilities, thus contributing to glutamate excitotoxicity. Ultimately, chronic expression of these molecules and the continuous neuronal damage, coupled with a dysfunctional glymphatic system, release uncleared proteins into the CSF and the lymph nodes, where they can be surveilled by immune cells. Figure created with https://BioRender.com. AD, Alzheimer's disease; CSF, cerebrospinal fluid; GFAP, glial fibrillary acidic protein

AD is not traditionally considered an autoimmune disease. There is growing evidence, however, suggesting that immune system dysregulation and co‐existing central nervous system (CNS) inflammation may play a role in the development and progression of AD.^27^, ^28^ Research has shown that chronic immune dysregulation in the brain, mediated by immune cells and inflammatory molecules in the CNS, contributes to the neurodegenerative process that is invariably seen in Alzheimer's disease (Figure 1) and other neurodegenerative disorders.^28^, ^29^ Some studies have identified immune‐related genetic variants, which are associated with an increased risk of developing AD.^30^, ^31^ Furthermore, the brain's immune system response, with part of it being the neuroinflammatory response, implicates the activation of microglia and astrocytes, which are specialized immune cells in the CNS.^28^ Dysregulation of this immune response can contribute to the neuronal damage and the cognitive decline seen in AD.

Although AD is not a typical autoimmune disorder, understanding the role of immune system dysfunction in the pathogenesis of the disease is an area of active research. Therapeutic approaches targeting immune‐mediated mechanisms may hold promise for the future treatment and prevention of AD. Already, recent data support the view that new therapeutics, such as the glucagon‐like peptide‐1 (GLP‐1) receptor agonist semaglutide, and similarly acting drugs, is a class of medications known to reduce inflammation, and may reduce AD and other neurodegenerative disease risk,^32^ in addition to other diseases that are also aggravated by inflammation. Thus, uncovering the putative autoimmune components of AD (Table S1) may lead to the development of new concepts for AD pathogenesis, diagnosis, and therapy.

#### 1.2.3 Susceptibility loci for late‐onset AD and relation to (auto)immunity

In addition to deposits of amyloid plaques and neurofibrillary tangles (NFTs), growing evidence demonstrates that complex and multifaceted biological processes can arise during AD pathogenesis. Numerous research directions, including genome‐wide associations, clinical correlation, and mechanistic studies, have pointed to a potential autoimmune contribution to AD pathology. Below, we briefly present published data suggesting associations between autoimmunity and AD and demonstrate the need for new approaches and novel laboratory techniques to further characterize and validate potential brain‐specific autoantibodies.

Numerous susceptibility loci for late‐onset AD (LOAD) were identified in published GWAS studies.^33^ In 2013, Lambert et al. conducted a two‐stage meta‐analysis of GWAS that had studied individuals of European ancestry. In the first stage, they used genotyped and imputed data from four previously published GWAS data sets consisting of over 17,000 AD cases and 37,000 controls. In the second stage, more than 11,000 SNPs were tested for association in an independent set of AD cases and controls. In the combined first and second stages, 19 loci reached significance, including the apolipoprotein E (*APOE)* locus (apolipoprotein E) and *SORL1* (sortilin‐related receptor 1). Out of those, 11 loci were novel, among them *PTK2B* (protein tyrosine kinase 2β) and human leukocyte antigen (*HLA)‐DRB5‐DRB1* (major histocompatibility complex, class II, DRβ5 and DRβ1). PTK2B is known to regulate the humoral immune response, the inflammasome response^34^ and microglial activation that is Aβ‐dependent,^35^ and its mRNA levels have been implicated in systemic lupus erythematosus (SLE).^36^ The aforementioned HLA alleles have been found to influence the risk of acquiring several autoimmune diseases, including multiple sclerosis (MS) and SLE.^37^

More recent meta‐analyses by Shade et al. and Bellenguez et al. revealed more loci with significance (*P < 2×10^−8^)*, including *TREM2*, *PIK3R5*, *LZTS1*, *SORT1*, and *ADAM17*.^38^, ^39^ In the first instance, a two‐stage GWAS on 111,326 AD and 677,663 controls discovered 75 total and 42 new risk loci. Through pathway enrichment analysis, they found an involvement of those risk loci with Aβ/tau, endocytosis, and immune pathways, including microglial activation.^39^ Additionally, LZTS1 is associated with rheumatoid arthritis (RA), SORT1 with MS and the processing of antigens in dendritic cells, and ADAM17 with the release of soluble TNF‐α, as seen in RA and SLE.^40^, ^41^, ^42^, ^43^ Mutations in *TREM2*, a gene expressed in microglia that modulates microglial activity, have consistently been shown to increase the risk of developing AD.^30^ Although not validated, this observation reinforces microglial endocytosis of Aβ in AD. In the second instance, 7,804 participants were included.^38^ The role of these newly identified genetic loci in the development of LOAD continues to be under investigation.

Adding to the genetic predisposition of AD, Cornejo‐Olivas et al. have identified a Peruvian family in which there were four AD cases as well as two individuals with cognitive impairment.^44^ In total, six siblings had cognitive impairment in a single family. These patients had heterozygous loss‐of‐function mutations in *SORL1*, coding for a protein that is involved in the trafficking of Aβ from cellular membranes to inside the cells for degradation. Microglia express SORL1, and loss of function of this gene in human induced pluripotent stem cell (hiPSC) ‐derived microglia‐like cells led to impaired lysosomal exocytosis, a process that is involved in immune response.^45^

### 1.3 Brain inflammation and autoimmunity in diseases other than AD

Exploring autoimmune‐related neurological diseases beyond AD provides critical insights into the possible underlying mechanisms of immune dysregulation in the brain, including a broader context for understanding autoimmunity in AD.

In addition to AD, autoantibodies found in patients’ cerebrospinal fluid (CSF) or serum can cause other diseases, which are collectively known as autoimmune encephalitides, as well as other CNS disorders.^46^ These diverse maladies are characterized by the presence of specific autoantibodies and various symptoms, including personality changes, loss of memory, psychosis, etc.

The most common autoantibodies encountered so far are those against N‐methyl‐D‐aspartate receptor (NMDAR), causing anti‐NMDAR encephalitis^47^, and autoantibodies against leucine‐rich‐glioma‐inactivated (anti‐LG11) antibody, causing anti‐LG11 encephalitis.

**FIGURE 2:**
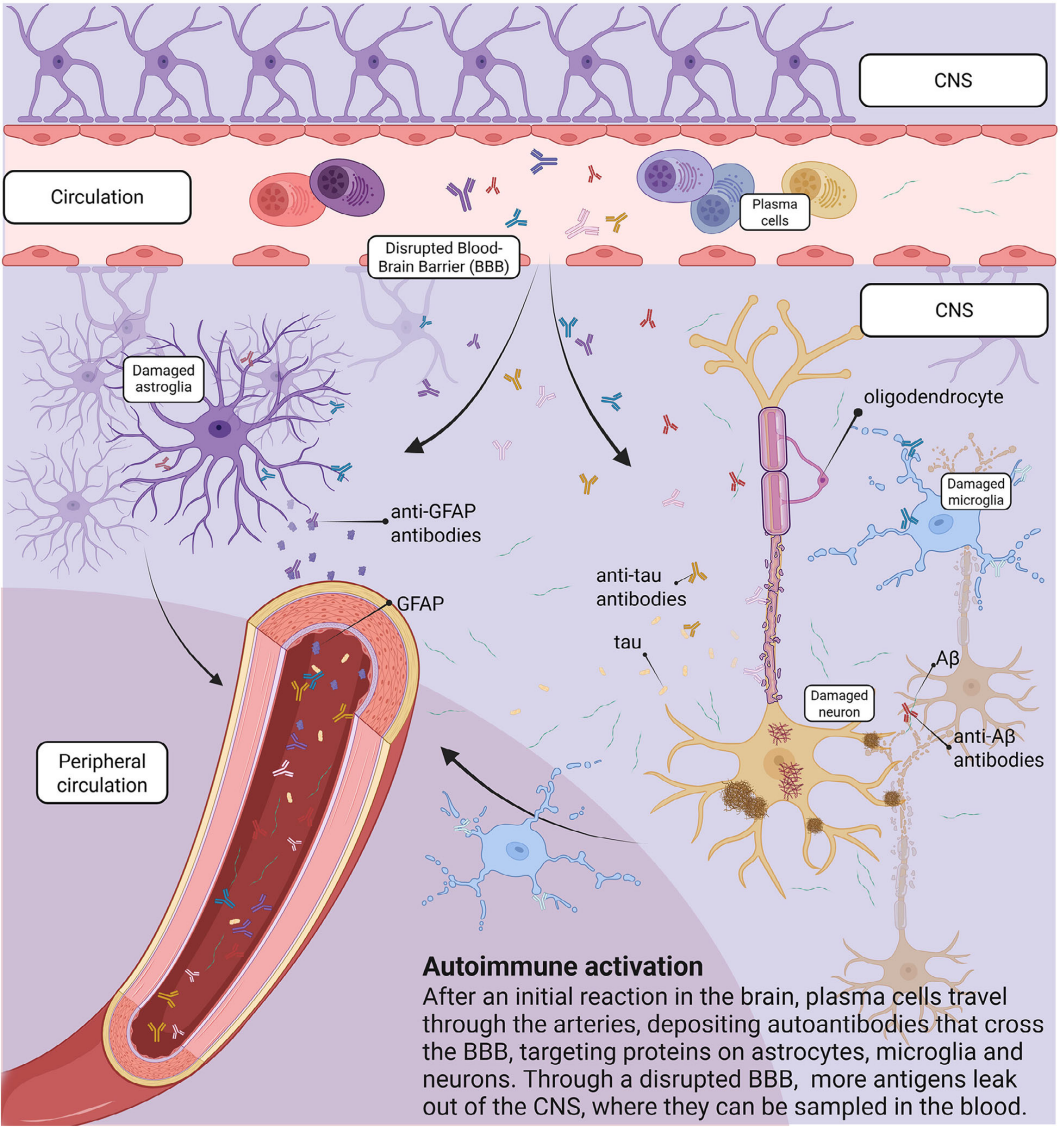
The autoimmune activation that occurs in the CNS leaks into the periphery through a hypothesized disrupted BBB. Examples include autoantibodies to GFAP, Aβ, and tau, among others that have been identified multiple times and are described in Tables 4 and 5, and Table S2. Although not validated in AD, anti‐GFAP antibodies have been shown to induce an autoimmune disease called “autoimmune GFAP astrocytopathy,” where GFAP autoantibodies target astrocytes, causing astrogliosis and inflammation. Image created in https://BioRender.com. AD, Alzheimer's disease; BBB, blood–brain barrier; CNS, central nervous system; GFAP, glial fibrillary acidic protein

These autoantibodies either originate in the brain or from immune cells in the periphery and enter the brain through the bloodstream via blood–brain barrier (BBB) dysfunction that increases permeability. (BBB dysfunction is currently debated in the field, so we will treat it as a hypothesis herein.) (Figure 2). Under normal conditions, the BBB acts as a selectively permeable membrane that regulates the exchange of molecules and cells between the CNS and peripheral blood circulation.^48^ In AD, BBB is hypothesized to precede or exacerbate inflammatory processes through immune component infiltration into the CNS.^49^, ^50^, ^51^ Autoantibody generation requires T‐cell–B‐cell interaction (Figure 1). For more information, see ref [52]. Autoantibodies can then bind to targets on the surface of neurons or at synapses, disrupting myelination and causing axonal damage. This immune‐mediated damage may be further aggravated by iron deposition.^53^, ^54^ Excess iron promotes oxidative stress through reactive oxygen species formation, leading to synaptic dysfunction.^53^, ^54^ These mechanisms form a complex interplay between immune dysregulation, impaired vascular integrity, and neurodegeneration—one that may not only involve passive leakage, but also active immune surveillance at the CNS border.

Recently, Fitzpatrick et al. identified lymphoid structures in the dura mater, which they termed the rostral‐rhinal venolymphatic hub.^55^ After challenge with systemic or nasal antigens, these lymphoid hubs generate plasma cells, which support humoral immunity by production of antibodies and/or autoantibodies. This finding is of profound importance because it demonstrates that immune cells residing in the meninges can drive autoimmunity via plasma cell differentiation and autoantibody production—supporting the broader argument that the brain hosts numerous immune cells capable of initiating autoimmune reactions.

Other research efforts aim to identify autoantibodies that might have a role in a wider range of psychiatric disorders. Whereas, as mentioned, high levels of some autoantibodies cause encephalitis, at lower levels, the same, or other, autoantibodies might cause chronic psychiatric illness in a much larger population. For example, in people with depression, Pruss and colleagues found autoantibodies that target astrocytes, the most abundant cell in the central nervous system.^56^, ^57^ The same group described autoantibodies against certain cells in the hippocampus, isolated from the CSF of a young woman with obsessive–compulsive disorder. Other, presumably autoantibody‐mediated neuropsychiatric disorders have implicated autoantibodies against potassium voltage‐gated channel subfamily A Member 2 (KCNA2). Autoantibodies against voltage‐gated potassium channels were also found in the CSF of patients with AD.^58^

### 1.4 Autoantibodies in AD

Our general hypothesis for this review is that AD pathogenesis may be partially driven by aberrant autoimmune reactions, whereby autoantibodies target self‐proteins in the brain, progressively leading to demyelination and axonal damage. Similarly, Jaycox et al. and others have expanded on this hypothesis to speculate that autoantibodies influence a range of conditions beyond AD and autoimmune diseases.^4^ The already existing and rapidly expanding literature on the topic is an indication of its scientific and clinical importance.^4^, ^59^, ^60^, ^61^

We compiled published literature supporting the view that autoimmunity may be one of the main drivers of immune system‐linked neuronal damage in AD. One of our major goals was to compare the suggested candidate pathogenetic autoantibodies between various studies, as one way of verifying the robustness and the reproducibility of the relevant published findings.

We are aware that to compile a meaningful comparative list from diverse publications, critical clinical and methodological differences between reports need to be carefully taken into consideration to avoid bias.^62^, ^63^ Otherwise, the identified differences may gear toward misleading conclusions regarding the identity and pathobiology of the putative, and presumably offending autoantibodies. Such differences include, but are not limited to, testing sample selection (serum vs CSF), heterogeneous populations of patients and controls, target antigen selection (brain‐specific versus non‐brain‐specific proteins), and the technical assay performance for autoantibody identification, including assay sensitivity and specificity (Table 3). With these caveats in mind, we first considered the role of autoantibodies against Aβ and tau.

#### 1.4.1 Naturally occurring antibodies and pathogenic autoantibodies

Naturally occurring antibodies (NAbs) are present in the body from birth, with poly‐reactivity and high‐binding avidity,^64^ though their numbers decrease with age.^65^ Through their low‐to‐moderate affinity, they provide homeostatic and housekeeping functions to the immune system, as well as help with the clearance of cellular debris.^64^ During sustained dyshomeostatic conditions, such as chronic inflammation, these low‐binding NAbs can trigger affinity maturation in various immune cells, causing the production of high‐binding affinity immunoglobulin (Ig) Gs that target self‐antigens.^66^ In these cases, development of pathogenic autoantibodies (PAbs) may occur.

In other cases, dysregulation of self‐tolerance mechanisms, such as negative selection of autoreactive lymphocytes, can trigger an autoimmune reaction and disease. Cross reactivity from molecular mimicry can also produce PAbs (Figure 1), and an example of that comes from immune reactivity against *Streptococcus pyogenes* and the subsequent erroneous attack against cardiac valves, leading to rheumatic endocarditis.^67^ Finally, epitope spreading and antigen‐presenting cell dysregulation can trigger additional activation of autoreactive B cells.^68^

Both NAbs and PAbs originate through V(D)J recombination (variable, diversity, joining) in the developing B‐cells, which creates unique variable regions of the heavy and light chains.^69^, ^70^ Following B‐cell activation, these variable regions undergo somatic hypermutation in the germinal centers, increasing antigen‐binding affinity. Importantly, unlike NAbs, PAbs show high levels of somatic hypermutation, hinting at affinity maturation and a selection for self‐antigen recognition.^71^ Finally, PAbs undergo class switching, often to an IgG isotype, whereas NAbs are more likely to be of the IgM isotype.^72^

In this review, we focus on AD‐related PAbs, as well as NAbs to a lesser extent, and do not provide detailed accounts of other dementias or psychiatric disorders, although it has been reported that some of the same autoantibodies found in AD are also found in other neurodegenerative diseases.^73^, ^74^

#### 1.4.2 NAbs and PAbs to Aβ and tau and their association with cognitive decline and cerebral amyloidosis in AD

The relevance of NAbs to Aβ (NAbs‐Aβ) in AD pathogenesis remains unclear,^75^ although these NAbs were discovered almost 35 years ago.^76^ Interestingly, despite the reported autoantibodies against Aβ and tau having been termed NAbs, no studies have analyzed the nucleic acid sequences to determine whether they are NAbs or PAbs.^64^ We can thus hypothesize that these authors categorized them as NAbs based on their existence and higher concentrations in the healthy control population. Below, we will describe some of the recent research on NAbs‐Aβ and PAbs‐Aβ.

In 2009, Britschgi and colleagues discovered that certain amyloidogenic fragments of Aβ generated high immunoreactivities, specifically oligomers and those with post‐translational modifications.^77^, ^78^ Most importantly, Aβ29‐35 fragments had significantly higher immunoreactivity in non‐cognitively impaired controls (NCIs) than the AD group. This was validated by a recent paper, where Liu et al. aimed to investigate NAbs‐Aβ levels, the associations with Aβ burden, epitope specificity, and cognitive decline cross‐sectionally and longitudinally^79^ (Table 4). NAbs‐Aβ levels in plasma and CSF were analyzed according to the position of their epitopes. NAbs‐Aβ targeting the N‐terminus of Aβ increased, and those targeting the mid‐domain of Aβ decreased in both CSF and plasma in AD patients compared to controls, and this was confirmed by peptide microarrays. These results aligned with the established knowledge since the 1980s, that the Aβ N‐terminus is the immunodominant part of the peptide.^80^, ^81^ N‐terminus‐specific NAbs‐Aβ increase correlated with worsening cognition, as did the decrease of the mid‐domain‐specific NAbs‐Aβ in AD, but not in controls. This was repeated 2 years later by the Liu group.^82^

**TABLE 4:** Autoantibodies against the two pathologic hallmarks of AD, Aβ, and tau.

First author, year, autoantibody	Total sample size (*n*)	Methodology, biofluid, validation (*y*/*n*)	Autoantibody in AD (↑/↓)	Value (Dx, Px, Tx, Pxv)	Epitopes analyzed, isotype
***Aβ autoantibodies***					
Terryberry, 1998, PAbs‐Aβ^83^	*N* = 418	Method: ELISA Serum, CSF Val: N	Increase	Dx	Aβ1‐42, IgG
Hyman, 2001, NAbs‐Aβ^74^	*N* = 353	Method: ELISA Plasma Val: N	No difference	No value	Soluble Aβ1‐42, Ig
Du, 2001, NAbs‐Αβ^84^	*N* = 96	Method: ELISA, IP assay CSF, plasma	Decrease	Unknown	(Aβ25‐35^**^, Aβ1‐40, Aβ1‐42), IgG
Weksler, 2002, NAbs‐Aβ^85^	*N* = 78	Method: ELISA Serum Val: N	Decrease	Dx	Soluble Aβ1‐42, IgG, and total Ig
Nath, 2003, PAbs‐Aβ^86^	*N* = 95	Method: ELISA Serum, CSF Val: N	Increase	Dx	Soluble and aggregated Aβ1‐42, IgG
Gruden, 2004, PAbs‐Aβ^87^	*N* = 32	Method: ELISA Serum Val: N	Increase	Dx	IC^#^, Aggregated Aβ25–35^**^, IgG, IgM, IgA
Baril, 2004, NAbs‐ Aβ^88^	*N* = 70	Method: ELISA Serum Val: N	No difference	No value	(Aβ1–40, Aβ1 –42), IgG1,
Mruthinti, 2004, PAbs‐Aβ^89^	*N* = 75	Method: ELISA, Plasma Val: N	Increase	Dx	(Aβ1‐42, Aβ1‐40), IgG
Moir, 2005, NAbs‐ Aβ^90^	*N* = 118	Method: Sandwich ELISA Plasma Val: N	No difference (monomer), Decrease (oligomer)	Pxv, Tx	(Low molecular mass oligomeric cross‐linked and monomer Aβ1‐42, Aβ1‐40), IgG
Brettschneider, 2005, NAbs‐Aβ^91^	*N* = 126	Method: IP assay Serum Val: N	Decrease	Dx	Aβ1‐42, Ig
Jianping, 2006, NAbs‐Aβ^92^	*N* = 40	Method: ELISA Serum Val: N	Decrease	Dx	Aβ1‐40, IgG
Gruden, 2007, PAbs‐Aβ^93^	*N* = 76	Method: ELISA, dot blot Serum Val: N	Increase and decrease in later stages	Dx	Aβ25–35^**^, IgG
Song, 2007, NAbs‐Aβ^94^	*N* = 346	Method: ELISA Serum Val: N	Decrease	Dx	Aβ1‐42, IgG
Gustaw, 2008, NAbs‐Aβ^95^	*N* = 103	Method: ELISA Serum Val: Y	Increase following antibody‐antigen dissociation	Dx, Tx	Aβ1‐42, IgG
Xu, 2008, NAbs‐Aβ^96^	*N* = 268	Method: plaque immunoreactivity Plasma Val: N	No difference	No value	(Aβ1‐40, Aβ1‐42), IgG
Britschgi, 2009, NAbs‐Αβ^77^	*N* = 218	Method: Western blots, Peptide microarrays, ELISA Plasma and CSF Val: Y	Decrease	Tx	(Aβ3(pE)‐42, Aβ11(pE)‐42, Aβ29‐M35 (MetSox)^**^, etc.), IgG
Sohn, 2009, NAbs‐Aβ^97^	*N* = 346	Method: ELISA Serum Val: N	Decrease	Dx	Aβ1‐42, IgG
Gustaw‐Rothenberg, 2009, NAbs‐Aβ^98^	*N* = 70	Method: ELISA Serum Val: Validation study of^95^	Increase	Dx	Aβ1‐42, IgG
Storace, 2010, PAbs‐ Aβ IC^†^, NAbs‐Aβ^99^	*N* = 285	Method: ELISA Plasma Val: N	Increase	Px	Aβ1‐42, IgG
Klaver, 2011, NAbs‐Aβ^100^	*N* = 30	Method: ELISA Serum Val: N	Non‐significant increase	Unknown	Soluble oligomer and monomer Aβ1‐42, non‐IgG specific
Maftei, 2013, NAbs‐Aβ immune complex^101^	*N* = 112	Method: Sandwich ELISA Serum, CSF Val: N	Increase	Px	Aβ21–37^**^, IgG
Qu, 2014, NAbs‐Aβ^102^	*N* = 113	Method: ELISA, dot‐blot Serum Validation: Y	Decrease	Dx over 65 years of age	(Aβ1‐15^*^, Aβ16‐30^**^, Aβ31‐42^***^, Aβ1‐42), IgG2
Vojdani, 2018, NAbs‐Aβ and against other antigens^103^	*N* = 94	Method: ELISA Serum Val: N	Increase	Dx, Tx	Aβ1‐42, IgG
Liu, 2021, NAbs‐Aβ^79^	*N* = 182	Method: ELISA, peptide microarrays Plasma, CSF Val: Y	Increase in Aβ1‐12 and Aβ7‐18, decrease in Aβ19‐30 and Aβ25‐36	Px, Tx	(Aβ1‐12^*^, Aβ7‐18^*^, Aβ13‐24^**^, Aβ19‐30^**^, Aβ25‐36^**^, Aβ31‐42^***^, Aβ1‐42), IgG2
Monteiro, 2022, NAbs‐Αβ^104^	*N* = 5	Method: cyclic voltammetry, atomic force microscopy CSF, plasma Val: N	Increase	Dx	Aβ1‐40, IgG
Renuka Sanotra, 2022, NAbs‐Aβ, NAbs‐Aβ‐HNE^105^	*N* = 235	Method: ELISA Serum Val: N	Increase	Dx	(Aβ1‐16^*^, Aβ7‐28^**^, IgG, IgM
Xu, 2023, NAbs‐Aβ^82^	*N* = 80	Method: ELISA CSF, plasma	Decrease in Mid‐domain peptides, increase in N‐terminus peptides	Dx	(Aβ1‐12^*^, Aβ7‐18^*^, Aβ13‐24^**^, Aβ19‐30^**^, Aβ25‐36^***^, Aβ31‐42^***^, Aβ1‐42), IgG
Knecht, 2024, NAbs‐Aβ^106^	*N* = 235	Method: ELISA Plasma Val: N	Decrease (IgA) compared to DLB Increase (IgG) compared to NCIs	Dx for subgroup	Aβ1‐42, IgG
***Tau autoantibodies***					
Terryberry, 1998, PAbs‐tau (MAPT)^83^	*N* = 418	Method: ELISA Serum, CSF Val: N	Not detected	No value	‐, IgG
Rosenmann, 2006, NAbs‐tau (MAPT)^107^	*N* = 59	Method: ELISA Serum, CSF Val: N	Increase (IgM) for p‐tau, No difference (IgG)	Unknown	(P‐tau at residues 195‐213, p‐tau phosphorylated at 202/205), IgG, IgM
Bartos, 2012, NAbs‐tau (MAPT)^108^	*N* = 80	Method: ELISA Serum, CSF Val: N	Increase	Unknown	‐, IgG
Klaver, 2017, NAbs‐tau (MAPT)^109^	*N* = 30	Method: ELISA Serum Val: N	No difference in AD Increased in MCI (p‐tau IgG)	Unknown	(Truncated tau 196‐207, p‐tau199, p‐tau202), IgG, IgM
Krestova, 2018, NAbs‐tau (MAPT)^110^	*N* = 134	Method: ELISA Serum, CSF Val: N	No significant difference, some sex difference but minimal	No value	(Truncated tau 155‐421, bovine tau, tau 1‐441), IgG
Bartos, 2018, NAbs‐tau (MAPT)^111^	*N* = 100	Method: ELISA Serum Val: N	Decrease	Dx, Tx	‐, IgG
Yu, 2020, NAbs‐tau (MAPT)^112^	*N* = 284	Method: ELISA, Immunofluorescence, Western blot Plasma Val: Y	No difference	No value	Tau441, IgG
Fang, 2024, PAbs‐tau (MAPT)^113^	*N* = 1686	Method: ELISA Serum Val: Y	Increase	Dx	MAPT502‐758 (Clone number 09F)
Knecht, 2024, NAbs‐tau (MAPT)^106^	*N* = 235	Method: ELISA Plasma Val: N	Increase (IgA) compared to DLB Decrease (IgG) compared to PD	Dx for subgroup	‐, IgG, IgA, IgM

* N‐terminus peptide.

** Mid‐domain peptide.

*** C‐terminus peptide.

^†^ IC = immune complex.

Abbreviations: AD, Alzheimer's disease; CSF, cerebrospinal fluid; DLB; Dx, diagnostic; ELISA, enzyme‐linked immunosorbent assay; MAPT, microtubule‐associated protein tau; MCI, mild cognitive impairment; Nabs, naturally occurring antibodies; Pabs, pathogenic autoantibodies; Px, prognostic; Pxv, predictive; Tx, therapeutic.

PAbs‐Aβ, NAbs‐Aβ, NAbs‐tau, and PAbs‐tau in serum/plasma and CSF have been discovered and extensively analyzed using a number of methodologies (Table 4). Most importantly, these types of discoveries have led to the development of passive immunotherapy as an AD therapeutic. The first monoclonal antibody to be approved by the Food and Drug Administration (FDA) was Aducanumab,^114^ which targeted the N‐terminus of Aβ. By taking NAbs‐Aβ and the B‐cells producing them from healthy elderly people, they reverse‐engineered this seemingly protective autoantibody to finally create a commercial product.^115^ Subsequent clinical trials yielded more of these drugs, with Lecanemab by Eisai and Donanemab by Eli Lilly being the most successful thus far and gaining United States FDA approval for treatment of patients with MCI and early AD.^116^, ^117^

The administration of these monoclonal antibody‐based therapies, however, can also lead to rare focal inflammation, either with edema (amyloid‐related imaging abnormalities‐edema [ARIA‐E]), and cerebral microhemorrhage (ARIA‐H).^10^ Careful consideration and precision medicine can reduce these incidences. For example, these ARIAs are thought to result from an immune activation around Αβ plaques, and some patients are more likely to exhibit them if they have pre‐existing cardiovascular conditions or have a higher Αβ42/total tau index ratio in CSF.^118^ These patient‐specific factors will help us tailor treatment approaches that reduce ARIA risks.

The microtubule‐associated protein tau (*MAPT*) gene and its protein product tau are linked to AD and other neurodegenerative diseases.^119^ The most established hypothesis posits that hyperphosphorylation of tau is an important pathological hallmark of AD and the building block of NFTs.^112^ Hyperphosphorylation at serine, threonine, and tyrosine residues reduces tau's microtubule binding affinity, leading it to detach and accumulate in the cytoplasm, where structural changes make it more prone to aggregating into NFTs.^120^ Alternative hypotheses suggest that post‐translational modifications or in cellular stress conditions, tau can spontaneously aggregate into filaments and later undergo hyperphosphorylation as a response to aggregation.^120^

Although we speculate that tau movement from the brain to the blood circulation has the potential to stimulate the production of PAbs against tau (PAbs‐tau),^113^ most studies have investigated NAbs to tau (NAbs‐tau) in plasma, and whether they are altered in AD patients, by using immunofluorescence staining, Western blot assays, and ELISA^108^, ^111^, ^112^, ^121^ (Table 4). These data suggest the existence of NAbs‐tau in human blood from an early age,^121^ with an enrichment toward IgG_3_ and low avidity. It has been found that truncated tau forms demonstrated the highest avidity and reactivity.^112^, ^122^

The plasma concentration of NAbs‐tau in adult NCIs and AD patients was also analyzed by ELISA. No significant difference in the plasma levels of NAbs‐tau was observed between NCI and AD groups,^112^ and other studies have corroborated this, or have found no existence of such autoantibodies in patients with AD.^83^, ^107^, ^109^ Furthermore, the plasma levels of NAbs‐tau had no significant correlation with the Mini‐Mental State Examination (MMSE) scores. One group found decreased levels of Nabs‐tau in serum and increased levels in the CSF, but no other data suggested this to be true.^108^, ^111^

From these analyses, it can be concluded that IgG NAbs‐tau likely exist in human blood, but contrary to PAbs‐Aβ and Nabs‐Aβ do not seem to differ in concentration between the NCI and AD groups. It remains unclear whether certain isotypes are more pathogenic and have clinical value in AD.

#### 1.4.3 NAbs and PAbs to other antigen targets in AD

We conducted an earlier review of the literature to identify autoantibodies that could be associated with AD pathobiology.^60^ Our previous survey identified 84 putative autoantibodies, which were present in either serum/plasma or CSF. By merging these data with additional/subsequent studies, we developed a more comprehensive list with 123 autoantibodies (Table S2). As already mentioned, the significant clinical and technical differences between the examined studies do not allow for unbiased comparisons of the findings. To discern if the already identified autoantibodies in AD are likely to be true positives or false positives, we compared how frequently the same autoantibodies were independently identified in the published studies and by how many different research groups. The most frequently identified AD‐related autoantibodies are those shown separately in Table 5. Below, we examine some recently performed analyses for autoantibody discovery.

**TABLE 5:** Consistently discovered autoantibodies in AD, in independent studies

Autoantibodies against protein	Protein found in CSF proteome/ brain tissue		Protein implicated in neurodegeneration	Frequency of autoantibody reporting in literature^*^	Reference
MAPT (IgG)	No	Yes	Yes^123^	Often (n > 5)	^83^, ^100^, ^107^, ^110^, ^111^, ^113^
Aβ (IgG	Yes	Yes	Yes	Often (n > 10)	^77^, ^79^, ^93^, ^96^, ^97^, ^98^, ^100^, ^101^, ^102^, ^103^, ^104^
AT1R (AGTR1 (IgG)	Yes (n.v.^†^)	Yes	Yes^124^	Twice	^125^, ^126^
DNAJC8 (IgG	No	Yes	Yes^127^	Twice	^113^, ^128^
ITPR1 (IgG	Yes (n.v.)	Yes	Yes^129^	Twice	^6^, ^130^
AGER (RAGE (IgG)	No	No	Yes^131^	Sometimes (n = 4)	^89^, ^113^, ^132^, ^133^
MAP4 (IgG	Yes	Yes	Unknown	Twice	^5^, ^134^
S100b (IgG	Yes	Yes	Yes^135^	Twice	^93^, ^136^
GFAP (IgG, IgM	Yes	Yes	Yes^137^	Sometimes (n = 4)	^83^, ^136^, ^138^, ^139^
NFH (IgG	Yes (n.v.)	Yes	Yes^140^	Twice	^83^, ^111^
NMDAR (IgA, IgM, IgG	No	Yes	Yes^141^	Twice	^142^, ^143^
BP180 (IgG	Yes (n.v.)	No	Yes^144^	Twice	^145^, ^146^
GM1 (IgM, IgG	Yes	Yes	Yes^147^	Sometimes (n = 3)	^148^, ^149^, ^150^

^†^ n.v . = non‐validated.

* Autoantibody studies published by the same group were not counted in the frequency.

Abbreviations: AD, Alzheimer's disease; CSF, cerebrospinal fluid; GFAP, glial fibrillary acidic protein; Ig, immunoglobulin; MAPT, microtubule‐associated protein tau; NFH, heavy neurofilament; NMDAR, N‐methyl‐D‐aspartate receptor.

Gu et al. examined the association of 28 NAbs in plasma against antigens stemming from AD‐associated genes found in previous GWAS, and found that 17 of them showed decreased NAbs in patients with AD compared to NCIs and patients with MCI^151^ (Table 4). Some of those antigens included TREM2, SORL1, ADAM10, and CLU (Table S2). Although the authors report the autoantibodies to be of the NAbs kind, evidence for the claim is not provided. These data, if correct, suggest that a number of NAbs are produced against proteins that take part in processes related to AD, and that during disease pathogenesis, a decrease in their concentration occurs, either as a result of the disease, as a causative agent in the disease, or as a result of aging. Such a decrease could lead to loss of immune regulation and homeostasis, and impaired Aβ clearance, potentially contributing to chronic inflammation and accumulation of toxic aggregates.^75^, ^152^ Interestingly, MCI patients had the lowest amounts of these autoantibodies. Unfortunately, the authors do not disclose whether these MCI patients progressed to AD, and thus, we cannot make conclusions about the exaggerated decrease of the autoantibodies and whether they are drivers of neurodegeneration.

Sera from 236 subjects (AD, MCI, NCI, Parkinson's disease, MS, breast cancer) were screened with protein microarrays to identify potential biomarkers for MCI patients that are suspected to progress to AD.^153^ The performance of the markers was evaluated using machine learning and statistical methods, including random forest plots and ROC curves. Their autoantibody biomarker panels could differentiate MCI patients from the other groups with an overall claimed accuracy, sensitivity, and specificity of 100%. Importantly, they were capable of differentiating MCI patients from those with mild‐moderate AD and other neurological and non‐neurological controls with high accuracy, but were not able to be used effectively to discriminate between disease stages that did not exhibit substantial site‐ or pathology‐specific differences. These seemingly impressive data need independent confirmation and comparison to well‐recognized blood‐based AD biomarkers.^153^, ^154^

Fang et al. used literature reports to compile a candidate list of 100 putative serum autoantibodies to antigens that are seen to be dysregulated in AD. From the list, seven candidate AD‐specific autoantibodies were identified, including MAPT, DNAJC8, KDM4D, SERF1A, CDKN1A, AGER, and ASXL1 (Table S2, Table 5).^113^ These top candidates were then used to create a classification model with high accuracy (AUC = 0.94), claiming that these autoantibodies could distinguish AD from other neurodegenerative diseases and showed better performance than Aβ and tau protein concentrations in CSF in predicting cognitive decline. Of note, no comparisons to contemporary blood‐based diagnostic assays were made.^113^ This study concluded that AD onset and progression are possibly accompanied by a yet unappreciated serum autoantibody response. Therefore, the authors suggested that this panel could be used as a biomarker for the early detection and progression of AD as well as a source of candidate therapeutic targets. Three of these putative antigens have previously been discovered by independent analyses and people (Table 5).

Shim et al. analyzed serum autoantibody profiles using the HuProt proteome microarray and a discovery set of NCIs (*n*  =  5) and AD (*n*  =  5) subjects.^155^ From the microarray, five autoantigens were selected for a validation experiment with NCIs (*n*  =  44), MCI (*n*  =  44), and AD (*n*  =  44) subjects using ELISA. Interestingly, although the results have not been replicated, they found that the serum levels of four autoantibodies, including anti‐ATCAY, HIST1H3F, NME7, and PAIP2 IgG, were significantly different among NCIs, MCI, and/or AD groups. Two of these markers, anti‐ATCAY and anti‐PAIP2 autoantibodies, exhibited significant correlation with neuropsychological scores such as MMSE.

By using a protein array of 1600 proteins, Wang et al. identified autoantibodies that were present exclusively in sera of people with dementia due to AD (*n* = 16) but not in the non‐AD dementia group (*n* = 24).^5^ Six autoantibodies were found: PANK3, PTP4A1, PIK3R1, NAP1L3, MAP4, and SOX2 (Table 2). MAP4 was seen in an independent analysis, increasing the possibility that, along with tau, this microtubule‐associated protein is implicated mechanistically in the disease.

Another group looked at autoantibodies with agonist function, which are also described in cardiovascular disorders, due to cardiovascular comorbidities existing in AD.^126^, ^156^ These authors investigated a potential association between antibodies to the angiotensin 2 type 1 receptor (anti‐AT1R) and AD. Samples from patients with mild AD (*n* = 92) and NCIs (*n*  =  102) were included, and ELISA was used to measure anti‐AT1R in serum. They found that AD patients had significantly higher levels of anti‐AT1R compared with NCIs (*p*  =  0.04), and this difference was interestingly found only in patients without hypertension and diabetes. Anti‐AT1R has also been discovered by Wallukat et al., 3 years after the discovery by this group.^125^, ^126^

Our own analyses using a novel mass spectrometry method to explore the autoantibody repertoire in the CSF of patients with AD did not reproduce most of the previously found autoantibodies by others (Table S2).^6^ In a subsequent study (our data; not published), the previously found autoantibodies were not verified in another, independent patient set, supporting our already expressed concern that clinical and/or analytical variability may lead to significant false discovery.^63^, ^157^

Most recently, Liu et al. discovered autoantibodies against the beta‐site amyloid precursor protein‐cleaving enzyme 1 (BACE1), a hallmark enzyme that takes part in the Aβ‐generation pathway.^158^ By comparing the plasma of non‐AD dementia cases (*n* = 28), patients with AD (*n* = 86), preclinical AD (*n* = 169), and NCIs (*n* = 232), they found that PAbs‐BACE1 were highest in clinical AD patients, and that it exacerbated amyloid deposition in the brain, tau hyperphosphorylation and neurodegeneration. Although the in vivo effect of the PAbs‐BACE1 was observed in mice, it nevertheless supports the hypothesis of a pathogenic role of autoantibodies in the progression of AD.

### 1.5 Discussion of data presented related to reported autoantibodies

A sole pathological event leading to AD remains undiscovered, despite decades of intense research efforts. It is probable that the causes of AD are the result of a myriad of intertwining pathologies. However, hope remains that a single pathological event could lead to the many events observed in AD brain tissues, thereby catalyzing the parallel presentation of simultaneous pathologies. For example, age‐related vascular diseases or traumatic brain injuries, which include an impaired BBB, are a common denominator associated with various degrees of dementia.^159^, ^160^

It is clear from the above presentations that the existence of immune‐related abnormalities cannot be dismissed in AD. Although the presence of specific NAbs and PAbs is not clinically validated, they may present another way of Aβ removal through immune complex formation,^161^ leading to microglial activation and phagocytosis. This mechanism, if activated, mimics vaccination efforts for immunologically removing Aβ by microglial cells using Aβ autoantibodies and maintaining homeostatic conditions. It may be possible to use other strategies, in addition to Aβ monoclonal antibodies, to achieve better therapeutic benefit. It must be noted, however, that despite consistent identification of some of these autoantibodies in AD (Table 5), the role of these autoantibodies in disease initiation and progression has not been unequivocally demonstrated, whether it be causal, a response to cellular damage, an epiphenomenon, or a protective mechanism. For example, these autoantibodies may develop after disease initiation or upstream of Aβ accumulation, in response to increased immune system activity or another dysregulated pathway. Perhaps these autoantibodies are associated and represent sequelae of AD but may not be pathogenic per se. Distinguishing true PAbs from autoantibodies arising as a secondary to neurodegeneration will be of key importance to understanding the disease better and potentially discovering a predictive value in these markers. Animal studies, correlation studies between the level of neurodegeneration and autoantibody load, and temporal studies will help in identifying the role of autoantibodies in AD.

Several clinical trials that modulate microglia signaling pathways and microglial activation are currently underway (NCT06535308, NCT06489548, NCT05564169), and time will tell if these changes will be clinically significant. If significance is reached, it would highlight the potential role of NAbs in supporting Αβ clearance, and the possibility of selectively enhancing NAbs or suppressing PAbs for therapy.

### 1.6 True positives versus false positives and false negatives

Above, we have described numerous candidate autoantibodies in CSF and serum/plasma of AD patients (Tables 4 and 5, Table S2). We also stressed the possibility that these findings may represent false discovery and already mentioned several reasons for this (Table 3). The only way to confirm or refute the truth of our hypotheses is by conducting well‐designed validation studies after the establishment of specificity and sensitivity thresholds. These studies should be able to identify the autoantibodies at least three independent times using the same technique in the same laboratory.

Yet, by putting forward the criterion that any putative pathogenic antibody should be identified in different patient groups AND by different investigators (Table 5), we can speculate as to which of these autoantibodies may have priority for validation success in such confirmatory studies. Also, most published studies focus on the ability of these autoantibodies to separate patient groups (NCI, MCI, AD) rather than looking for mechanistic leads, which would further strengthen their involvement of these autoantibodies in AD pathobiology.

Below we selected some entries from Table 5 to add more mechanistic connections per the published literature.

Angiotensin II is a potent vasopressor hormone and a primary regulator of aldosterone secretion.^162^ It is an important effector controlling blood pressure and volume in the cardiovascular system,^162^ and has been implicated as an effector of oxidative stress.^163^ Finally, activation of AT1R has been linked to cognitive impairment, and AD has previously been linked to high blood pressure^163^, ^164^; thus, there may be a functional link between autoantibodies and AD.^125^, ^126^

DnaJ Heat Shock Protein Family (Hsp40) Member C8 (DNAJC8) is one of several autoantibodies that may be associated with AD.^113^, ^128^ A study that measured autoantibodies in serum samples found that DNAJC8, along with other autoantibodies, could distinguish AD from other neurodegenerative diseases^113^ (Table 5). The study also found that these autoantibodies could predict cognitive decline better than Aβ and tau protein concentrations in CSF, but mechanistic studies linking DNAJC8 to AD have not been published.^113^ Since DNAJC8 is a HSP70 chaperone linked to mitochondrial function, and mitochondrial function is central to AD pathogenesis,^165^, ^166^ it is possible that this autoantibody relates to AD pathobiology.

Our own studies identified autoantibodies to inositol 1,4,5‐triphosphate receptor type 1 (ITPR1) in CSF of AD patients, among other autoantibodies.^6^ ITPR1 is expressed in hippocampal and cortical neurons, and ITPR1 autoantibodies have been found to be involved in cognitive decline.^167^, ^168^ Disturbance in ITPR1 function can lead to dysfunctional calcium signaling, and studies have shown that Aβ1‐42 can dysregulate ITPR1.^168^ These additional functional characteristics qualify ITPR1 as a good target for validation studies.

The advanced glycosylation end product receptor (RAGE) is a multiligand receptor and a member of the immunoglobulin family of cell surface receptors.^169^ It interacts with other molecules implicated in development and inflammation; for example, RAGE–ligand interactions result in NF‐κB activation. In addition, RAGE is also implicated in certain diseases, particularly in AD.^169^ Various isoforms of the RAGE gene have been described through alternatively spliced transcript variants, as well as non‐protein‐coding variants, some of which have been seen to interact with Aβ.^169^

The protein encoded by microtubule‐associated protein 4 (MAP4) is a major non‐neuron‐specific microtubule‐associated protein that localizes in neuronal dendrites.^170^ Similar to MAPT, MAP4 is composed of a microtubule‐binding domain and a project domain, with multiple isoforms existing,^170^ and it promotes microtubule assembly. MAP4 was shown to be N‐homocysteinylated and to accumulate in protein NFTs and aggregates.^171^ This could potentially explain how autoantibodies against MAP4 were found to be increased in both AD and MCI patients, and that they were associated with tauopathy.^5^, ^134^

### 1.7 The way forward

In this review, we can make the case that AD can now be diagnosed more easily and earlier with blood‐based testing.^172^, ^173^ These tests have good sensitivity and specificity. Blood‐based tests for AD, quantifying the ratio of plasma phosphorylated tau 217 (p‐tau217) relative to non‐p‐tau217, expressed as percentage of p‐tau217, combined with the amyloid‐β 1‐42 and amyloid‐β 1‐40 plasma ratio (the amyloid probability score 2 [APS2]) identified AD in primary care and secondary care provided superior performance compared with the diagnostic accuracy after standard clinical evaluation (not using AD biomarkers).^172^

Current and future approaches targeting the immune system, applied early during disease diagnosis, may make the difference for either prevention or for slowing/stopping disease progression. Sarazin et al. recently suggested additional approaches to AD immunotherapy strategies, which are currently focusing on single‐agent immunity through amyloid‐removing antibodies such as lecanemab and donanemab. They suggest identifying targets that include both central and peripheral immunity, such as general neuroinflammation suppressors (Ozempic), immunomodulatory therapies, vaccination, etc.^174^ Like modern and relatively effective cancer combination therapies, similar approaches could be tried for AD, with a goal to reduce or arrest neuronal damage by mounting attacks to supress both peripheral and CNS immunity.

Passive immunotherapy is currently at the forefront of AD therapeutics, with multiple clinical trials underway to evaluate drug efficacy and safety in preclinical disease (NCT04468659).^175^ The future of immunotherapy in AD, however, is likely to incorporate active vaccination approaches.^176^ These strategies will aim to stimulate the immune system to mount a long‐lasting immune response against Αβ or tau.

Recent developments in immunotherapy have also sought to cure autoimmune diseases (such as type‐1 diabetes, SLE, and MS) by suppressing the patient's entire immune response.^177^ This is an effective strategy, but people who are treated have an elevated risk of infections and cancer. An alternative strategy would be to engineer cells that can dampen the immune response, increasing the threshold of immunologic tolerance. If, indeed, enhanced autoimmunity is a feature of AD,^178^ then inducing some level of tolerance to certain antigens in the brain, or other manipulations that strengthen immune responses (e.g., vaccinations), which induce autoantibody responses, may have therapeutic value. For example, CAR T‐cells (chimeric autoantibody receptor T‐cell therapies) could be engineered to recognize autoantibodies on B‐cells, thus facilitating the destruction of these autoantibody‐producing B‐cells. Intuitively, then, both immune system activators and suppressors may find their way in AD future therapeutics. Unfortunately, slow recruiting and difficulties in finding eligible patients have hindered clinical trials in the field of immunomodulation (e.g., NCT03132272, NCT03179501).

We have not been the first to draw attention to the autoimmune landscape in AD, nor will we be the last. The quest to uncover immune‐related connections between autoantibodies and AD spans at least 37 years, with the earliest studies having reported brain‐associated and neuron‐binding autoantibodies in AD.^76^, ^83^, ^179^, ^180^, ^181^, ^182^

We may ask why are we still skeptical about the implications of humoral immunity in this disease? Perhaps, the first question that needs to be answered pertains to the almost universal existence of brain‐reactive autoantibodies in human serum,^183^ and the role of humoral immunity in controlling the development and progression of a great variety of neurodegenerative diseases. A deep understanding of how active vaccines and other immunotherapies can integrate with the humoral and cellular immune environment will be pivotal. No doubt, this better understanding will facilitate new ways of preventing, stop the progression, or even reversing this new epidemic that threatens to surpass cancer as the top reason for morbidity and mortality of people of all ages, but primarily the elderly.

## CONFLICT OF INTEREST STATEMENT

The authors declare no conflict of interest. Author disclosures are available in the Supporting Information.

## CONSENT STATEMENT

Patient consent was not necessary.

## Supporting information



Supporting Information

Supporting Information

Supporting Information
