# Supplementary material for: The role of autoantibodies in Alzheimer's disease: Pathogenetic connections or epiphenomena?
Source: Alzheimers Dement. 2025 Jul 22;21(7):e70484. doi: 10.1002/alz.70484 (PMC12284324; doi:10.1002/alz.70484)
Supplement: Supplementary file 2 — Supporting Information [file ALZ-21-e70484-s003.docx]

**Supplementary Table 1.** Partial list of links between immunity and AD.

| **Link** | **Comments** |
| --- | --- |
| GWAS (1,2) | GWAS revealed that certain genotypes of the HLA system confer higher risk for developing AD (such as HLA-DRB5-DRB1). |
| Immune system dysregulation (3–5) | It has been demonstrated that there is co-existence of immune system dysregulation and chronic inflammation within the brain of AD patients, as well in peripheral tissues. |
| Role of microglia (6–8) | In AD, microglia can become overactivated, producing inflammatory markers and activating components of the innate immune system, leading to subsequent neuronal damage. |
| Glymphatic system (9,10) | Through this system, neuronal antigens can drain into cervical lymph nodes, promoting autoimmune reactions. Decreased drainage may facilitate increased amyloid beta deposition, neurofibrillary tangle formation and accumulation of neurotoxins that trigger the immune system. |
| Blood-Brain Barrier (BBB) (11) | Breakdown of BBB through aging and damaged vasculature, allows an increased number of immune cells and pathogens to gain access to the brain parenchyma, potentially increasing autoimmune reactivity. *Topic is of high debate following concerns of scientific misconduct and data falsification* (12)*.* |
| Cytotoxic autoantibodies (13–16) | Presence of autoantibodies against amyloid beta deposits and tau is well established. Cytotoxic autoantibodies in CSF or serum, against a plethora of other targets has been described. Their pathogenetic role is speculative at present. |
| Adaptive immune cells (17) | Adaptive immune cells (CD4+ and CD8+ T cells) are localized close to neurons, and they can upregulate chemokine receptors and chemokines (CXCR4-CXCL12, interleukin 17A), ultimately causing neuroaxonal damage. |
| Infectious agents (18–20) | Infectious agents (such as HSV-1, P gingivalis, H pylori) may trigger abnormal amyloid precursor protein (APP) processing and increased amyloid beta deposition; they can induce autoimmunity through molecular mimicry. *Validation is ongoing.* |
| Gut microbiome (21) | Gut microbiota products may be able to enter the brain and induce an immune reaction through molecular mimicry. *Validation is ongoing.* |
| Lung microbiome (22) | Lung microbiome modulates the activity of immune cells in the brain (microglia), increasing neuroinflammatory components and inducing autoimmunity. |
| Astrocytic immune memory (23,24) | Astrocytes can acquire epigenetic immune memory that amplifies astrocyte’s proinflammatory signalling in response to specific molecular stimuli and during autoimmune diseases. |
| Mitochondrial activity (25) | Innate and adaptive immune system activation is sustained in part by activated microglia through mitochondrial complex 1 activity. These microglial cells can produce neuronal neurotoxins such as TNFs, interleukins, nitric oxide and reactive oxygen species which are causing failure of remyelination and neuronal/axonal damage. |

References

1. Mathys H, Davila-Velderrain J, Peng Z, Gao F, Mohammadi S, Young JZ, et al. Single-cell transcriptomic analysis of Alzheimer’s disease. Nature. 2019 Jun;570(7761):332–7.

2. Listì F, Candore G, Balistreri CR, Grimaldi MP, Orlando V, Vasto S, et al. Association between the HLA-A2 allele and Alzheimer disease. Rejuvenation Res. 2006;9(1):99–101.

3. Leng F, Edison P. Neuroinflammation and microglial activation in Alzheimer disease: where do we go from here? Nat Rev Neurol. 2021 Mar;17(3):157–72.

4. Altmann DM. Neuroimmunology and neuroinflammation in autoimmune, neurodegenerative and psychiatric disease. Immunology. 2018;154(2):167–8.

5. Cao W, Zheng H. Peripheral immune system in aging and Alzheimer’s disease. Mol Neurodegener. 2018 Oct 3;13(1):51.

6. Shi Q, Gutierrez RA, Bhat MA. Microglia, Trem2, and Neurodegeneration. Neuroscientist. 2024 May 20;10738584241254118.

7. Ryu JK, McLarnon JG. A leaky blood–brain barrier, fibrinogen infiltration and microglial reactivity in inflamed Alzheimer’s disease brain. J Cell Mol Med. 2009;13(9a):2911–25.

8. Yang YM, Shang DS, Zhao WD, Fang WG, Chen YH. Microglial TNF-α-dependent elevation of MHC class I expression on brain endothelium induced by amyloid-beta promotes T cell transendothelial migration. Neurochem Res. 2013 Nov;38(11):2295–304.

9. Louveau A, Plog BA, Antila S, Alitalo K, Nedergaard M, Kipnis J. Understanding the functions and relationships of the glymphatic system and meningeal lymphatics. J Clin Invest. 2017;127(9):3210–9.

10. Da Mesquita S, Louveau A, Vaccari A, Smirnov I, Cornelison RC, Kingsmore KM, et al. Functional aspects of meningeal lymphatics in ageing and Alzheimer’s disease. Nature. 2018 Aug;560(7717):185–91.

11. Sweeney MD, Sagare AP, Zlokovic BV. Blood–brain barrier breakdown in Alzheimer disease and other neurodegenerative disorders. Nat Rev Neurol. 2018 Mar;14(3):133–50.

12. Piller C. Science. 2024 [cited 2024 Dec 2]. Top Alzheimer’s researcher goes ‘on leave’ amid misconduct concerns. Available from: https://www.science.org/content/article/top-alzheimer-s-researcher-goes-leave-amid-misconduct-concerns

13. Levin EC, Acharya NK, Han M, Zavareh SB, Sedeyn JC, Venkataraman V, et al. Brain-reactive autoantibodies are nearly ubiquitous in human sera and may be linked to pathology in the context of blood–brain barrier breakdown. Brain Research. 2010 Jul 23;1345:221–32.

14. Fang L, Jiao B, Liu X, Wang Z, Yuan P, Zhou H, et al. Specific serum autoantibodies predict the development and progression of Alzheimer’s disease with high accuracy. Brain Behav Immun. 2024 Jan;115:543–54.

15. Wu J, Li L. Autoantibodies in Alzheimer’s disease: potential biomarkers, pathogenic roles, and therapeutic implications. J Biomed Res. 2016 Sep;30(5):361–72.

16. Chatanaka MK, Sohaei D, Diamandis EP, Prassas I. Beyond the amyloid hypothesis: how current research implicates autoimmunity in Alzheimer’s disease pathogenesis. Crit Rev Clin Lab Sci. 2023 Sep;60(6):398–426.

17. Gate D, Tapp E, Leventhal O, Shahid M, Nonninger TJ, Yang AC, et al. CD4+ T cells contribute to neurodegeneration in Lewy body dementia. Science. 2021 Nov 12;374(6569):868–74.

18. Shiota S, Murakami K, Yoshiiwa A, Yamamoto K, Ohno S, Kuroda A, et al. The relationship between Helicobacter pylori infection and Alzheimer’s disease in Japan. J Neurol. 2011 Aug;258(8):1460–3.

19. Itzhaki RF. Herpes simplex virus type 1 and Alzheimer’s disease: possible mechanisms and signposts. FASEB J. 2017 Aug;31(8):3216–26.

20. Ryder MI. Porphyromonas gingivalis and Alzheimer disease: Recent findings and potential therapies. J Periodontol. 2020 Oct;91(Suppl 1):S45–9.

21. Khan MF, Wang H. Environmental Exposures and Autoimmune Diseases: Contribution of Gut Microbiome. Front Immunol. 2020;10.

22. Hosang L, Canals RC, van der Flier FJ, Hollensteiner J, Daniel R, Flügel A, et al. The lung microbiome regulates brain autoimmunity. Nature. 2022 Mar;603(7899):138–44.

23. Sofroniew MV. Astrocyte cells in the brain have immune memory. Nature. 2024 Mar;627(8005):744–5.

24. Lee HG, Rone JM, Li Z, Akl CF, Shin SW, Lee JH, et al. Disease-associated astrocyte epigenetic memory promotes CNS pathology. Nature. 2024 Mar;627(8005):865–72.

25. Peruzzotti-Jametti L, Willis CM, Krzak G, Hamel R, Pirvan L, Ionescu RB, et al. Mitochondrial complex I activity in microglia sustains neuroinflammation. Nature. 2024 Apr;628(8006):195–203.
