# Supplementary material for: The role of autoantibodies in Alzheimer's disease: Pathogenetic connections or epiphenomena?
Source: Alzheimers Dement. 2025 Jul 22;21(7):e70484. doi: 10.1002/alz.70484 (PMC12284324; doi:10.1002/alz.70484)
Supplement: Supplementary file 3 — Supporting Information [file ALZ-21-e70484-s002.docx]

**Supplementary Table 2. Complete list of reported autoantibodies in AD.**

| Autoantibodies against protein | Protein found in CSF proteome/ Brain tissue | | Protein implicated in neurodegeneration | Frequency of autoantibody reporting in literature | Refs |
| --- | --- | --- | --- | --- | --- |
| Aβ (IgG) | Yes | Yes | Yes (1) | Often (n>10) | (2–9) |
| CRYZ (IgG) | Yes | Yes | Yes (10) | Once | (11) |
| ATP2A2 (IgG) | Yes (n.v.) | Yes | Yes (12) | Once |  |
| SRPX (IgG) | Yes | Yes | Yes (13) | Once |  |
| WDR1 (IgG) | Yes | Yes | No (14) | Once |  |
| SLC3A2 (IgG) | Yes | Yes | Yes (15) | Once |  |
| PLD4 (IgG) | Yes | Yes | Yes (16) | Once |  |
| OMgp (IgG) | Yes | Yes | Yes (17) | Once |  |
| TIMP2 (IgG) | Yes | Yes | Yes (18) | Once |  |
| ASRGL1 (IgG) | Yes | Yes | Yes (19) | Once |  |
| hnRNPH (IgG) | Yes (n.v.) | Yes | Yes (20) | Once |  |
| SERPINE2 (IgG) | Yes | Yes | Yes (21) | Once |  |
| FMOD (IgG) | Yes | Yes | Unknown | Once |  |
| CBLN3 (IgG) | Yes | Yes | Unknown | Once |  |
| CTSF (IgG) | Yes | Yes | Yes (22) | Once |  |
| CDH13 (IgG) | Yes | Yes | Yes (23) | Once |  |
| ITPR1 (IgG) | Yes (n.v.) | Yes | Yes (24) | Twice | (11,25) |
| MAPT (IgG) | Yes | Yes | Yes (26) | Often (n>10) | (27,28,28–36) |
| DNAJC8 (IgG) | Yes | Yes | Yes (37) | Twice | (27,38) |
| KDM4D (IgG) | Yes (n.v.) | No | Possibly (39) | Once | (27) |
| SERF1A (IgG) | No | Yes | Yes (40) | Once |  |
| CDKN1A (IgG) | Yes (n.v.) | Yes | Yes (41) | Once |  |
| ASXL1 (IgG) | Yes (n.v.) | Yes | No (42,43) | Once |  |
| AGER (RAGE) (IgG) | Yes | Yes | Yes (44) | Sometimes (n=5) | (27,45–48) |
| ApoA1 (IgM) | Yes | Yes | Yes (49) | Once | (50) |
| ATCAY (IgG) | No | Yes | Unknown | Once | (51) |
| HIST1H3F (IgG) | Yes | Yes | Unknown | Once |  |
| NME7 (IgG) | Yes (n.v.) | Yes | Unknown | Once |  |
| PAIP2 (IgG) | Yes (n.v.) | Yes | Unknown | Once |  |
| SOS1 (IgG, IgM) | No | Yes | Yes (52) | Once | (53) |
| TNFRSF21 (IgG) | Yes | Yes | Yes (54) | Once |  |
| ATM (IgG) | Yes (n.v.) | Yes | Yes (55) | Once |  |
| S100A1 (IgG) | Yes | Yes | Yes (56) | Once |  |
| SP4 (IgM) | Yes (n.v.) | Yes | Yes (57) | Once |  |
| GNPAT (IgM) | Yes (n.v.) | Yes | Yes (58) | Once |  |
| CNTN2 (IgM) | Yes | Yes | Yes (59) | Once |  |
| PTCD2 (IgG) | No | Yes | Yes (60) | Once | (38) |
| FRMD8 (IgG) | Yes | Yes | Yes (61) | Once |  |
| C9orf9 (SPACA9) (IgG) | No | Yes | Unknown | Once |  |
| LGALS1 (IgG) | Yes | Yes | Yes (62) | Once |  |
| POMC, variant 2 (IgG) | Yes | Yes | Yes (63) | Once |  |
| MAPKAPK5, variant 1 (IgG) | Yes (n.v.) | Yes | Yes (64) | Once |  |
| CENTA2 (ADAP2) (IgG) | No | Yes | Yes (65) | Once |  |
| ANKHD1 (IgG) | Yes (n.v.) | Yes | Yes (66) | Once |  |
| MRPL34 (IgG) | No | Yes | No (67) | Once |  |
| MAP4 (IgG) | Yes | Yes | Unknown | Twice | (68,69) |
| NAP1L3 (IgG) | No | Yes | Yes (70) | Once | (68) |
| PANK3 (IgG) | Yes (n.v.) | Yes | Yes (71) | Once |  |
| PIK3R1 (IgG) | Yes (n.v.) | Yes | Yes (72) | Once |  |
| PTP4A1 (IgG) | Yes (n.v.) | Yes | Yes (73) | Once |  |
| SOX15 (IgG) | No | Yes | Unknown | Once |  |
| AT1R (AGTR1) (IgG) | Yes (n.v.) | Yes | Yes (74) | Twice | (75,76) |
| R-RAA aPLs* (IgG) | Yes | Yes | Yes | Once | (77) |
| ADAM10 (IgG) | Yes | Yes | Yes (78) | Once | (79) |
| ADAMTS1 (IgG) | Yes | Yes | Yes (80) | Once |  |
| CLU (IgG) | Yes | Yes | Yes (81) | Once |  |
| FERMT2 (IgG) | Yes (n.v.) | Yes | Yes (82) | Once |  |
| NDUFAF6 (IgG) | Yes (n.v.) | Yes | Yes (83) | Once |  |
| OARD1 (IgG) | Yes | Yes | Yes (84) | Once |  |
| PTK2B (IgG) | Yes (n.v.) | Yes | Yes (85) | Once |  |
| SLC24A4 (IgG) | Yes (n.v.) | Yes | Yes (86) | Once |  |
| SORL1 (IgG) | Yes | Yes | Yes (78) | Once |  |
| SPPL2A (IgG) | Yes (n.v.) | Yes | Yes (87) | Once |  |
| TREM2 (IgG) | Yes | Yes | Yes (78) | Once |  |
| WWOX (IgG) | Yes (n.v.) | Yes | Yes (88) | Once |  |
| ADAMTS4 (IgG) | Yes | Yes | Yes (80) | Once |  |
| SPRED2 (IgG) | Yes (n.v.) | Yes | Yes (89) | Once |  |
| TMEM163 (IgG) | Yes (n.v.) | Yes | Yes (90) | Once |  |
| TSPAN14 (IgG) | Yes (n.v.) | Yes | Yes (91) | Once |  |
| VKORC1 (IgG) | Yes (n.v.) | Yes | Yes (92) | Once |  |
| S100b (IgG) | Yes | Yes | Yes (93) | Twice | (31,94) |
| 5-HT (IgG) | Yes | Yes | Yes (95) | Twice (same group) | (31,96) |
| DA (IgG) | Yes | Yes | Yes (97) | Twice (same group) |  |
| Glutamate (IgG) | - | - | Yes (98) | Once | (99) |
| Tubulin (IgG) | Yes | Yes | Yes (100) | Once | (101) |
| GFAP (IgG, IgM) | Yes | Yes | Yes (102) | Sometimes (N=4) | (94,101,103,104) |
| ARHGAP26 (IgG) | Yes (n.v.) | Yes | Yes (105) | Once | (104) |
| KCNA2 (IgG) | Yes (n.v.) | Yes | Yes (106) | Once |  |
| Caspr2 (IgG) | Yes | Yes | Yes (107) | Once |  |
| GlyR (IgG) | No | Yes | Yes (108) | Once |  |
| GABA_B_R (IgG) | Yes | Yes | Yes (109) | Once |  |
| NFH (IgG) | Yes (n.v.) | Yes | Yes (110) | Sometimes (2/3 same group) | (28,101,111) |
| BACE1 (IgG) | Yes | Yes | Yes (112) | Once | (113) |
| PSEN1 (IgG) | Yes (n.v.) | Yes | Yes (114) | Once | (115) |
| Bim (IgG) | Yes (n.v.) | Yes | Yes (116) | Once | (117) |
| VGlut2 | No | Yes | Yes (118) | Once | (119) |
| IgLON5 | Yes | Yes | Yes (120) | Once | (121) |
| LGI1 | Yes | Yes | Yes (122) | Once |  |
| NMDAR (IgA, IgM, IgG) | No | Yes | Yes (123) | Sometimes (N=3, 2/3 same group) | (121,124,125) |
| p75ECD (IgG) | Yes | Yes | Yes (126) | Once | (127) |
| Neurochondrin (IgG) | No | Yes | Yes (128) | Once | (129) |
| IVD (IgG) | No | Yes | Unknown | Once | (130) |
| CYFIP1 (IgG) | Yes (n.v.) | Yes | Possibly (131) | Once |  |
| ADD2 (IgG) | Yes (n.v.) | Yes | Yes (132) | Once |  |
| BP180 (IgG) | Yes (n.v.) | No | Yes (133) | Sometimes (N=3, 2/3 same group) | (134–136) |
| α1-AR (IgG) | Yes (n.v.) | Yes | Yes (137) | Twice (same group) | (138,139) |
| Β2-AR (IgG) | Yes (n.v.) | Yes | Yes (140) | Once | (139) |
| MOG (IgG, IgM) | Yes | Yes | Yes (141) | Once | (142) |
| MAG (IgG) | Yes | Yes | Yes (143) | Once |  |
| PLP (IgG, IgM) | Yes (n.v.) | Yes | Yes (144) | Once |  |
| MBP (IgG, IgM) | Yes (n.v.) | Yes | Yes (145) | Twice | (142,146) |
| CHRNA7 (173-193) | Yes (n.v.) | Yes | Yes (147) | Once | (148) |
| GM1 (IgM, IgG) | Yes | Yes | Yes (149) | Sometimes (N=4, 2/4 same group) | (150–153) |
| GD1b (IgM) | Yes | Yes | Yes (149) | Once | (150) |
| GD (IgM) | Unknown | Yes | Yes (149) | Once | (152) |
| GA1 (IgM) | Unknown | Yes | Yes (149) | Once |  |
| GQ1bα (IgM) | Unknown | Yes | Yes (149) | Once |  |
| GQ1b (IgM) | Yes | Yes | Yes (149) | Once |  |
| GT1b (IgM, IgG) | Yes | Yes | Yes (149) | Once |  |
| GM3 (IgM, IgG) | Yes | Yes | Yes (149) | Once |  |
| α-synuclein (IgG) | Yes | Yes | Yes (154) | Once | (155) |
| Galanin (IgG) | Yes | Yes | Yes (156) | Once | (157) |
| α-MSH (IgG) | Yes (n.v.) | Yes | Possibly (158) | Once |  |
| SRPK1 (IgG) | Yes (n.v.) | Yes | Yes (159) | Once | (160) |
| ATP synthase β subunit (IgG) | Yes | Yes | Yes (161) | Once | (162) |
| PC* (IgM) | Yes (n.v.) | Yes | Yes (163) | Once | (164) |
| CCP (IgG) | No | Yes | Unkown | Once | (165) |
| OxLDL (IgG, IgM) | Yes | Yes | Yes (166) | Once | (167) |
| N-Hcy* (IgG) | Yes | Yes | Yes (168) | Once | (169) |
| NUPR1 (IgG) | Yes (n.v.) | Yes | Yes (170) | Once | (171) |
| OR8J1(IgG) | No | No | Unknown | Once |  |
| PYGB (IgG) | Yes (n.v.) | Yes | Yes (172) | Once |  |
| ANTXR1 (IgG) | Yes | Yes | Unknown | Once |  |

* Not a protein

n.v.= non-validated

CSF protein existence data was retrieved from: https://proteomics.uib.no/csf-pr-id/

Brain tissue existence data was retrieved from: https://www.proteinatlas.org/

**Abbreviations**: 5-HT, 5-hydroxytryptamine; α1-AR, alpha 1 adrenergic receptor; α-MSH, alpha-melanocyte-stimulating hormone; β2-AR, beta 2 adrenergic receptor; ADAM10, disintegrin and metalloproteinase domain-containing protein 10; ADAMTS1, a disintegrin-like and metalloproteinase with thrombospondin type 1 motifs; ADAMTS4, a disintegrin-like and metalloproteinase with thrombospondin type 4 motifs**;** ADAP2, ArfGAP with dual PH domains 2; ADD2, beta-adducin; AGER, advanced glycosylation end product-specific receptor; ANKHD1, Ankyrin repeat and KH domain containing 1; ANTXR1, anthrax toxin receptor 1; ApoA1, apolipoprotein A1; ARHGAP26, Rho GTPase activating protein 26; ASRGL1, isoaspartyl peptidase/L-asparaginase; ASXL1, polycomb group protein ASXL1; AT1R, Angiotensin 2 type 1 receptor; ATCAY, caytaxin; ATM, serine-protein kinase ATM; ATP2A2, sarcoplasmic/endoplasmic reticulum calcium ATPase 2; BACE1, beta-site amyloid precursor protein (APP)-cleaving enzyme 1; Bim, Bcl-2-interacting mediator of cell death; BP180, bullous pemphigoid 180; C9orf9, chromosome 9 open reading frame 9; Caspr2, contactin-associated protein-like 2; CBLN3, cerebellin-3; CCP, cyclic citrullinated peptide; CDH13, cadherin-13; CDKN1A, cyclin-dependent kinase inhibitor 1; CENTA2, Centaurin, alpha 2; CHRNA7,neuronal acetylcholine receptor alpha 7 protein fragment; CLU, clusterin; CNTN2, Contactin-2; CRYZ, quinone oxidoreductase; CTSF, cathepsin F; CYFIP1, cytoplasmic FMR1 interacting protein 1; DNAJC8, DnaJ homolog subfamily C member 8; DA, dopamine; FERMT2, fermitin family homolog 2; FMOD, fibromodulin; FRMD8, FERM domain containing 8; GA1, ganglioside GA1; GABA_B_R, gamma-aminobutyric acid B receptor; GD, ganglioside GD; GD1b, ganglioside GD1b; GlyR, glycine receptor; GM1, ganglioside GM1; GM3, ganglioside GM3; GNPAT, dihydroxyacetone phosphate acyltransferase; GQ1b, ganglioside GQ1b; GQ1bα, ganglioside GQ1bα; GT1b; ganglioside GT1b; HIST1H3F, histone H3.1; HNE adducts, 4-hydroxynonenal adducts; hnRNPH, heterogeneous nuclear ribonucleoprotein H; IgLON5, Ig-like domain-containing protein family member 5; ITPR1, inositol 1,4,5-trisphosphate-gated calcium channel ITPR1; IVD, isovaleryl-CoA-dehydrogenase; KCNA2, potassium voltage-gated channel subfamily A member 2; KDM4D, lysine-specific demethylase 4D; LGALS1, Galectin 1; LGI1, leucine-rich glioma inactivated protein 1; MAG, myelin-associated glycoprotein; MAP4, Microtubule associated protein 4; MAPT, microtubule-associated protein tau; MAPKAPK5, Mitogen-activated protein kinase-activated protein kinase 5; MBP, myelin basic protein; MOG, myelin oligodendrocyte glycoprotein; MRPL34, Mitochondrial ribosomal protein L34; N-Hcy, N-homocysteine; NAP1L3, Nucleosome assembly protein 1 like 3; NDUFAF6, NADH:Ubiquinone oxidoreductase complex assembly factor 6; NFH, neurofilament heavy polypeptide; NMDAR, N-methyl-D-aspartate receptor; NME7, nucleoside diphosphate kinase homolog 7; NUPR1, nuclear protein 1; OARD1, ADP-ribose glycohydrolase OARD1; OMgp, Oligodendrocyte-myelin glycoprotein; OR8J1, olfactory receptor 8J1; OxLDL, oxidized low-density lipoprotein; p75ECD, extracellular domain of p75 neurotrophin receptor; PAIP2, polyadenylate-binding protein-interacting protein 2; PANK3, Pantothenic acid kinase 3; PC, phosphorylcholine; PIK3R1, Phosphoinositide-3-kinase regulatory subunit 1; PLD4, 5'-3' exonuclease PLD4; PLP, proteolipoprotein; POMC, Proopiomelanocortin; PSEN1, presenilin 1; PTCD2, Pentatricopeptide repeat-containing protein 2; PTK2B, protein-tyrosine kinase 2-beta; PTP4A1, Protein tyrosine phosphatase type IVA Member 1; PYGB, glycogen phosphorylase ; R-RAA aPLs, Redox reactive antiphospholipid antibodies; S100A1, protein S100-A1; S100b, S100 calcium binding protein B; SERF1A, small EDRK-rich factor 1; SERPINE2, glia-derived nexin; SLC24A4, sodium/potassium/calcium exchanger 4; SLC3A2, amino acid transporter heavy chain SLC3A2; SORL1, sortilin-related receptor 1; SOS1, son of sevenless homolog 1; SOX15, (Sex Determining region Y)-Box 15; SP4, transcription factor Sp4; SPACA9; SPPL2A, signal peptide peptidase-like 2A; SPRED2, sprouty-related, EVH1 domain-containing protein 2; SRPK1, SR protein kinase 1; SRPX, sushi repeat-containing protein SRPX; TIMP2, metalloproteinase inhibitor 2; TMEM163, transmembrane protein 163 ; TNFRSF21, tumor necrosis factor receptor superfamily member 21; TREM2, triggering receptor expressed on myeloid cells 2; TSPAN14, tetraspanin-14 ; VGlut2; vesicular glutamate type 2; VKORC1, vitamin K epoxide reductase complex subunit 1; WDR1, WD repeat-containing protein 1; WWOX, WW domain-containing oxidoreductase.

**References**

1. Ashe KH. The biogenesis and biology of amyloid β oligomers in the brain. Alzheimer’s & Dementia. 2020;16(11):1561–7.

2. Moir RD, Tseitlin KA, Soscia S, Hyman BT, Irizarry MC, Tanzi RE. Autoantibodies to Redox-modified Oligomeric Aβ Are Attenuated in the Plasma of Alzheimer’s Disease Patients *. Journal of Biological Chemistry. 2005 Apr 29;280(17):17458–63.

3. Klaver AC, Coffey MP, Smith LM, Bennett DA, Finke JM, Dang L, et al. ELISA measurement of specific non-antigen-bound antibodies to Aβ1-42 monomer and soluble oligomers in sera from Alzheimer’s disease, mild cognitively impaired, and noncognitively impaired subjects. J Neuroinflammation. 2011 Aug 9;8:93.

4. Gustaw-Rothenberg K, Lerner A, Bonda DJ, Lee H gon, Zhu X, Perry G, et al. Biomarkers in Alzheimer’s disease: past, present and future. Biomark Med. 2010 Feb;4(1):15–26.

5. Gustaw KA, Garrett MR, Lee H gon, Castellani RJ, Zagorski MG, Prakasam A, et al. Antigen-Antibody Dissociation in Alzheimer Disease: A Novel Approach to Diagnosis. J Neurochem. 2008 Aug;106(3):1350–6.

6. Du Y, Dodel R, Hampel H, Buerger K, Lin S, Eastwood B, et al. Reduced levels of amyloid β-peptide antibody in Alzheimer disease. Neurology. 2001 Sep 11;57(5):801–5.

7. Baril L, Nicolas L, Croisile B, Crozier P, Hessler C, Sassolas A, et al. Immune response to Aβ-peptides in peripheral blood from patients with Alzheimer’s disease and control subjects. Neuroscience Letters. 2004 Jan 30;355(3):226–30.

8. Hyman BT, Smith C, Buldyrev I, Whelan C, Brown H, Tang MX, et al. Autoantibodies to amyloid-β and Alzheimer’s disease. Annals of Neurology. 2001;49(6):808–10.

9. Knecht L, Dalsbøl K, Simonsen AH, Pilchner F, Ross JA, Winge K, et al. Autoantibody profiles in Alzheimer´s, Parkinson´s, and dementia with Lewy bodies: altered IgG affinity and IgG/IgM/IgA responses to alpha-synuclein, amyloid-beta, and tau in disease-specific pathological patterns. J Neuroinflammation. 2024 Dec 3;21(1):317.

10. Yuhan L, Khaleghi Ghadiri M, Gorji A. Impact of NQO1 dysregulation in CNS disorders. Journal of Translational Medicine. 2024 Jan 2;22(1):4.

11. Lim B, Tsolaki M, Batruch I, Anastasiou A, Frontistis A, Prassas I, et al. Putative autoantibodies in the cerebrospinal fluid of Alzheimer’s disease patients. F1000Res. 2019 Nov 11;8:1900.

12. Goldberg J, Currais A, Ates G, Huang L, Shokhirev M, Maher P, et al. Targeting of intracellular Ca2+ stores as a therapeutic strategy against age-related neurotoxicities. npj Aging Mech Dis. 2020 Aug 24;6(1):1–9.

13. Inoue Y, Ueda M, Tasaki M, Takeshima A, Nagatoshi A, Masuda T, et al. Sushi repeat-containing protein 1: a novel disease-associated molecule in cerebral amyloid angiopathy. Acta Neuropathologica. 2017 Oct 1;134:1–13.

14. Wang J, Kou XL, Chen C, Wang M, Qi C, Wang J, et al. Hippocampal Wdr1 Deficit Impairs Learning and Memory by Perturbing F-actin Depolymerization in Mice. Cereb Cortex. 2019 Sep 13;29(10):4194–207.

15. Liu C, Li X, Li C, Zhang Z, Gao X, Jia Z, et al. SLC3A2 is a novel endoplasmic reticulum stress-related signaling protein that regulates the unfolded protein response and apoptosis. PLoS One. 2018 Dec 28;13(12):e0208993.

16. Singh S, Dransfeld U, Ambaw Y, Lopez-Scarim J, Farese RV, Walther TC. PLD3 and PLD4 synthesize S,S-BMP, a key phospholipid enabling lipid degradation in lysosomes. bioRxiv. 2024 Mar 21;2024.03.21.586175.

17. Koch JC, Tatenhorst L, Roser AE, Saal KA, Tönges L, Lingor P. ROCK inhibition in models of neurodegeneration and its potential for clinical translation. Pharmacology & Therapeutics. 2018 Sep 1;189:1–21.

18. Aksnes M, Capogna E, Vidal-Piñeiro D, Chaudhry FA, Myrstad M, Idland AV, et al. Matrix metalloproteinases are associated with brain atrophy in cognitively unimpaired individuals. Neurobiology of Aging. 2023 Nov 1;131:11–23.

19. Garcia-Montojo M, Fathi S, Rastegar C, Simula ER, Doucet-O’Hare T, Cheng YHH, et al. TDP-43 proteinopathy in ALS is triggered by loss of ASRGL1 and associated with HML-2 expression. Nat Commun. 2024 May 16;15:4163.

20. Conlon EG, Manley JL. RNA-binding proteins in neurodegeneration: mechanisms in aggregate. Genes Dev. 2017 Aug 1;31(15):1509–28.

21. Choi BH, Kim RC, Vaughan PJ, Lau A, Van Nostrand WE, Cotman CW, et al. Decreases in protease nexins in Alzheimer’s disease brain. Neurobiol Aging. 1995;16(4):557–62.

22. van der Zee J, Mariën P, Crols R, Van Mossevelde S, Dillen L, Perrone F, et al. Mutated CTSF in adult-onset neuronal ceroid lipofuscinosis and FTD. Neurol Genet. 2016 Sep 16;2(5):e102.

23. Patel PJ, Ren Y, Yan Z. Epigenomic analysis of Alzheimer’s disease brains reveals diminished CTCF binding on genes involved in synaptic organization. Neurobiology of Disease. 2023 Aug 1;184:106192.

24. Rosa N, Sneyers F, Parys JB, Bultynck G. Type 3 IP3 receptors: The chameleon in cancer. In: Spetz JKE, Galluzzi L, editors. International Review of Cell and Molecular Biology [Internet]. Academic Press; 2020 [cited 2024 Jul 16]. p. 101–48. (Cell Death Regulation in Health and Disease - Part A; vol. 351). Available from: https://www.sciencedirect.com/science/article/pii/S1937644820300083

25. Hansen N, Malchow B, Zerr I, Stöcker W, Wiltfang J, Timäus C. Neural cell-surface and intracellular autoantibodies in patients with cognitive impairment from a memory clinic cohort. J Neural Transm. 2021 Mar 1;128(3):357–69.

26. Barbier P, Zejneli O, Martinho M, Lasorsa A, Belle V, Smet-Nocca C, et al. Role of Tau as a Microtubule-Associated Protein: Structural and Functional Aspects. Front Aging Neurosci [Internet]. 2019 [cited 2022 Mar 30];11. Available from: https://www.frontiersin.org/article/10.3389/fnagi.2019.00204

27. Fang L, Jiao B, Liu X, Wang Z, Yuan P, Zhou H, et al. Specific serum autoantibodies predict the development and progression of Alzheimer’s disease with high accuracy. Brain Behav Immun. 2024 Jan;115:543–54.

28. Bartos A, Fialová L, Švarcová J. Lower Serum Antibodies Against Tau Protein and Heavy Neurofilament in Alzheimer’s Disease. J Alzheimers Dis. 2018 Jan 1;64(3):751–60.

29. Rosenmann H, Meiner Z, Geylis V, Abramsky O, Steinitz M. Detection of circulating antibodies against tau protein in its unphosphorylated and in its neurofibrillary tangles-related phosphorylated state in Alzheimer’s disease and healthy subjects. Neurosci Lett. 2006 Dec 20;410(2):90–3.

30. Krestova M, Ricny J, Bartos A. Changes in concentrations of tau-reactive antibodies are dependent on sex in Alzheimer’s disease patients. J Neuroimmunol. 2018 Sep 15;322:1–8.

31. Gruden MA, Davidova TB, Mališauskas M, Sewell RDE, Voskresenskaya NI, Wilhelm K, et al. Differential neuroimmune markers to the onset of Alzheimer’s disease neurodegeneration and dementia: Autoantibodies to Aβ(25–35) oligomers, S100b and neurotransmitters. J Neuroimmunol. 2007 May 1;186(1):181–92.

32. Britschgi M, Olin CE, Johns HT, Takeda-Uchimura Y, LeMieux MC, Rufibach K, et al. Neuroprotective natural antibodies to assemblies of amyloidogenic peptides decrease with normal aging and advancing Alzheimer’s disease. Proc Natl Acad Sci USA. 2009 Jul 21;106(29):12145–50.

33. Maftei M, Thurm F, Schnack C, Tumani H, Otto M, Elbert T, et al. Increased Levels of Antigen-Bound β-Amyloid Autoantibodies in Serum and Cerebrospinal Fluid of Alzheimer’s Disease Patients. PLoS One. 2013 Jul 18;8(7):e68996.

34. Vojdani A, Vojdani E. Amyloid-Beta 1-42 Cross-Reactive Antibody Prevalent in Human Sera May Contribute to Intraneuronal Deposition of A-Beta-P-42. Int J Alzheimers Dis. 2018;2018:1672568.

35. Yu ZY, Li WW, Yang HM, Mañucat-Tan NB, Wang J, Wang YR, et al. Naturally Occurring Antibodies to Tau Exists in Human Blood and Are Not Changed in Alzheimer’s Disease. Neurotox Res. 2020 Apr;37(4):1029–35.

36. Hromadkova L, Kolarova M, Jankovicova B, Bartos A, Ricny J, Bilkova Z, et al. Identification and characterization of natural antibodies against tau protein in an intravenous immunoglobulin product. J Neuroimmunol. 2015 Dec 15;289:121–9.

37. Ito N, Kamiguchi K, Nakanishi K, Sokolovskya A, Hirohashi Y, Tamura Y, et al. A novel nuclear DnaJ protein, DNAJC8, can suppress the formation of spinocerebellar ataxia 3 polyglutamine aggregation in a J-domain independent manner. Biochem Biophys Res Commun. 2016 Jun 10;474(4):626–33.

38. Nagele E, Han M, Demarshall C, Belinka B, Nagele R. Diagnosis of Alzheimer’s disease based on disease-specific autoantibody profiles in human sera. PLoS One. 2011;6(8):e23112.

39. Savchenko VL. Poly-ADP-ribosylation of KDM4D induces transcription in the hippocampus and amygdala [Internet]. 2024 [cited 2024 Jul 16]. Available from: https://www.researchsquare.com/article/rs-3909386/v1

40. Tsai TY, Chen CY, Lin TW, Lin TC, Chiu FL, Shih O, et al. Amyloid modifier SERF1a interacts with polyQ-expanded huntingtin-exon 1 via helical interactions and exacerbates polyQ-induced toxicity. Commun Biol. 2023 Jul 21;6(1):1–16.

41. Liu Q, Niu N, Wada Y, Liu J. The Role of Cdkn1A-Interacting Zinc Finger Protein 1 (CIZ1) in DNA Replication and Pathophysiology. International Journal of Molecular Sciences. 2016 Feb;17(2):212.

42. Matheus F, Rusha E, Rehimi R, Molitor L, Pertek A, Modic M, et al. Pathological ASXL1 Mutations and Protein Variants Impair Neural Crest Development. Stem Cell Reports. 2019 Apr 18;12(5):861–8.

43. Fujino T, Goyama S, Sugiura Y, Inoue D, Asada S, Yamasaki S, et al. Mutant ASXL1 induces age-related expansion of phenotypic hematopoietic stem cells through activation of Akt/mTOR pathway. Nat Commun. 2021 Mar 23;12(1):1826.

44. Kinscherf NA, Pehar M. Role and therapeutic potential of RAGE signaling in neurodegeneration. Curr Drug Targets. 2022;23(12):1191–209.

45. Mruthinti S, Schade RF, Harrell DU, Gulati NK, Swamy-Mruthinti S, Lee GP, et al. Autoimmunity in Alzheimer’s Disease as Evidenced by Plasma Immunoreactivity Against RAGE and Aβ42: Complication of Diabetes. Curr Alzheimer Res. 2006 Jul 1;3(3):229–35.

46. Mruthinti S, Buccafusco JJ, Hill WD, Waller JL, Jackson TW, Zamrini EY, et al. Autoimmunity in Alzheimer’s disease: increased levels of circulating IgGs binding Aβ and RAGE peptides. Neurobiol Aging. 2004 Sep 1;25(8):1023–32.

47. Mitchell MB, Buccafusco JJ, Schade RF, Webster SJ, Mruthinti S, Harrell DU, et al. RAGE and Abeta immunoglobulins: relation to Alzheimer’s disease-related cognitive function. J Int Neuropsychol Soc. 2010 Jul;16(4):672–8.

48. Wilson JS, Mruthinti S, Buccafusco JJ, Schade RF, Mitchell MB, Harrell DU, et al. Anti-RAGE and Abeta immunoglobulin levels are related to dementia level and cognitive performance. J Gerontol A Biol Sci Med Sci. 2009 Feb;64(2):264–71.

49. Tong J hui, Gong S qiang, Zhang Y song, Dong J ru, Zhong X, Wei M jie, et al. Association of Circulating Apolipoprotein AI Levels in Patients With Alzheimer’s Disease: A Systematic Review and Meta-Analysis. Front Aging Neurosci. 2022 May 18;14:899175.

50. Lin CY, Sheu JJ, Tsai IS, Wang ST, Yang LY, Hsu IU, et al. Elevated IgM against Nε-(Carboxyethyl)lysine-modified Apolipoprotein A1 peptide 141-147 in Taiwanese with Alzheimer’s disease. Clin Biochem. 2018 Jun;56:75–82.

51. Shim SM, Koh YH, Kim JH, Jeon JP. A combination of multiple autoantibodies is associated with the risk of Alzheimer’s disease and cognitive impairment. Sci Rep. 2022 Jan 25;12(1):1312.

52. Painter MM, Atagi Y, Liu CC, Rademakers R, Xu H, Fryer JD, et al. TREM2 in CNS homeostasis and neurodegenerative disease. Molecular Neurodegeneration. 2015 Sep 4;10(1):43.

53. Sim KY, Park SH, Choi KY, Park JE, Lee JS, Kim BC, et al. High-throughput epitope profiling of antibodies in the plasma of Alzheimer’s disease patients using random peptide microarrays. Sci Rep. 2019 Mar 14;9(1):4587.

54. Wiedemann C. Alzheimer’s protein in embryonic pruning. Nat Rev Neurosci. 2009 Apr;10(4):244–244.

55. Pizzamiglio L, Focchi E, Antonucci F. ATM Protein Kinase: Old and New Implications in Neuronal Pathways and Brain Circuitry. Cells. 2020 Aug 26;9(9):1969.

56. Cristóvão JS, Gomes CM. S100 Proteins in Alzheimer’s Disease. Frontiers in Neuroscience [Internet]. 2019 [cited 2024 Oct 9];13. Available from: https://www.ncbi.nlm.nih.gov/pmc/articles/PMC6532343/

57. Boutillier S, Lannes B, Buée L, Delacourte A, Rouaux C, Mohr M, et al. Sp3 and Sp4 Transcription Factor Levels Are Increased in Brains of Patients with Alzheimer’s Disease. Neurodegenerative Diseases. 2007 Oct 9;4(6):413–23.

58. Hossain MS, Mawatari S, Fujino T. Plasmalogens inhibit neuroinflammation and promote cognitive function. Brain Research Bulletin. 2023 Jan 1;192:56–61.

59. Chatterjee M, Del Campo M, Morrema THJ, de Waal M, van der Flier WM, Hoozemans JJM, et al. Contactin-2, a synaptic and axonal protein, is reduced in cerebrospinal fluid and brain tissue in Alzheimer’s disease. Alzheimers Res Ther. 2018 Jun 1;10:52.

60. Acharya NK, Nagele EP, Han M, Coretti NJ, DeMarshall C, Kosciuk MC, et al. Neuronal PAD4 expression and protein citrullination: Possible role in production of autoantibodies associated with neurodegenerative disease. Journal of Autoimmunity. 2012 Jun 1;38(4):369–80.

61. Li C, Wei Q, Hou Y, Lin J, Ou R, Zhang L, et al. Genome-wide analyses identify NEAT1 as genetic modifier of age at onset of amyotrophic lateral sclerosis. Molecular Neurodegeneration. 2023 Oct 23;18(1):77.

62. Chaudhary S, Chaudhary S, Rawat S, Kulkarni A, Bilgrami AL, Perveen A, et al. Galectins—Potential Therapeutic Targets for Neurodegenerative Disorders. Int J Mol Sci. 2022 Sep 20;23(19):11012.

63. Schneeberger M, Altirriba J, García A, Esteban Y, Castaño C, García-Lavandeira M, et al. Deletion of miRNA processing enzyme Dicer in POMC-expressing cells leads to pituitary dysfunction, neurodegeneration and development of obesity. Molecular Metabolism. 2013 Apr 1;2(2):74–85.

64. Kiddle SJ, Steves CJ, Mehta M, Simmons A, Xu X, Newhouse S, et al. Plasma protein biomarkers of Alzheimer’s disease endophenotypes in asymptomatic older twins: early cognitive decline and regional brain volumes. Transl Psychiatry. 2015 Jun;5(6):e584–e584.

65. Stricker R, Reiser G. Functions of the neuron-specific protein ADAP1 (centaurin-α1) in neuronal differentiation and neurodegenerative diseases, with an overview of structural and biochemical properties of ADAP1. Biol Chem. 2014 Nov 1;395(11):1321–40.

66. Vu L, Ghosh A, Tran C, Tebung WA, Sidibé H, Garcia-Mansfield K, et al. Defining the Caprin-1 Interactome in Unstressed and Stressed Conditions. J Proteome Res. 2021 Jun 4;20(6):3165–78.

67. Huang G, Li H, Zhang H. Abnormal Expression of Mitochondrial Ribosomal Proteins and Their Encoding Genes with Cell Apoptosis and Diseases. Int J Mol Sci. 2020 Nov 23;21(22):8879.

68. Wang BZ, Zailan FZ, Wong BYX, Ng KP, Kandiah N. Identification of novel candidate autoantibodies in Alzheimer’s disease. Eur J Neurol. 2020;27(11):2292–6.

69. Ehtewish H, Mesleh A, Ponirakis G, Lennard K, Hamad HA, Chandran M, et al. Profiling the autoantibody repertoire reveals autoantibodies associated with mild cognitive impairment and dementia. Frontiers in Neurology. 2023 Nov 30;14:1256745.

70. Xavier Garcia A, Xu J, Cheng F, Ruppin E, Schäffer AA. Altered gene expression in excitatory neurons is associated with Alzheimer’s disease and its higher incidence in women. Alzheimers Dement (N Y). 2023 Feb 8;9(1):e12373.

71. Hayflick SJ. Defective pantothenate metabolism and neurodegeneration. Biochem Soc Trans. 2014 Aug;42(4):1063–8.

72. Li H, Liu H, Lutz MW, Luo S, Alzheimer’s Disease Neuroimaging Initiative. Novel Genetic Variants in TP37, PIK3R1, CALM1, and PLCG2 of the Neurotrophin Signaling Pathway Are Associated with the Progression from Mild Cognitive Impairment to Alzheimer’s Disease. J Alzheimers Dis. 2023;91(3):977–87.

73. Noori A, Mezlini AM, Hyman BT, Serrano-Pozo A, Das S. Systematic review and meta-analysis of human transcriptomics reveals neuroinflammation, deficient energy metabolism, and proteostasis failure across neurodegeneration. Neurobiology of Disease. 2021 Feb 1;149:105225.

74. Labandeira‐Garcia JL, Parga JA. Nigral Neurons Degenerating in Parkinson’s Disease Express the Angiotensin Receptor Type 1 Gene. Mov Disord. 2022 Aug;37(8):1610–1.

75. Wallukat G, Prüss H, Müller J, Schimke I. Functional autoantibodies in patients with different forms of dementia. PLoS One. 2018 Mar 14;13(3):e0192778.

76. Giil LM, Kristoffersen EK, Vedeler CA, Aarsland D, Nordrehaug JE, Winblad B, et al. Autoantibodies Toward the Angiotensin 2 Type 1 Receptor: A Novel Autoantibody in Alzheimer’s Disease. J Alzheimers Dis. 2015 Jan 1;47(2):523–9.

77. McIntyre JA, Ramsey CJ, Gitter BD, Saykin AJ, Wagenknecht DR, Hyslop PA, et al. Antiphospholipid autoantibodies as blood biomarkers for detection of early stage Alzheimer’s disease. Autoimmunity. 2015;48(5):344–51.

78. Khezri MR, Mohebalizadeh M, Ghasemnejad-Berenji M. Therapeutic potential of ADAM10 modulation in Alzheimer’s disease: a review of the current evidence. Cell Communication and Signaling. 2023 Mar 14;21(1):60.

79. Gu D, Wang L, Zhang N, Wang H, Yu X. Decrease in naturally occurring antibodies against epitopes of Alzheimer’s disease (AD) risk gene products is associated with cognitive decline in AD. Journal of Neuroinflammation. 2023 Mar 15;20(1):74.

80. Gurses MS, Ural MN, Gulec MA, Akyol O, Akyol S. Pathophysiological Function of ADAMTS Enzymes on Molecular Mechanism of Alzheimer’s Disease. Aging Dis. 2016 Jan 11;7(4):479–90.

81. Foster EM, Dangla-Valls A, Lovestone S, Ribe EM, Buckley NJ. Clusterin in Alzheimer’s Disease: Mechanisms, Genetics, and Lessons From Other Pathologies. Front Neurosci [Internet]. 2019 Feb 28 [cited 2024 Dec 17];13. Available from: https://www.frontiersin.org/journals/neuroscience/articles/10.3389/fnins.2019.00164/full

82. Eysert F, Coulon A, Boscher E, Vreulx AC, Flaig A, Mendes T, et al. Alzheimer’s genetic risk factor FERMT2 (Kindlin-2) controls axonal growth and synaptic plasticity in an APP-dependent manner. Mol Psychiatry. 2021 Oct;26(10):5592–607.

83. Baide-Mairena H, Gaudó P, Marti-Sánchez L, Emperador S, Sánchez-Montanez A, Alonso-Luengo O, et al. Mutations in the mitochondrial complex I assembly factor NDUFAF6 cause isolated bilateral striatal necrosis and progressive dystonia in childhood. Mol Genet Metab. 2019 Mar;126(3):250–8.

84. Danhauser K, Alhaddad B, Makowski C, Piekutowska-Abramczuk D, Syrbe S, Gomez-Ospina N, et al. Bi-allelic *ADPRHL2* Mutations Cause Neurodegeneration with Developmental Delay, Ataxia, and Axonal Neuropathy. The American Journal of Human Genetics. 2018 Nov 1;103(5):817–25.

85. Guo Y, Sun CK, Tang L, Tan MS. Microglia PTK2B/Pyk2 in the Pathogenesis of Alzheimer’s Disease. Curr Alzheimer Res. 2023;20(10):692–704.

86. Yu L, Chibnik LB, Srivastava GP, Pochet N, Yang J, Xu J, et al. Association of Brain DNA Methylation in SORL1, ABCA7, HLA-DRB5, SLC24A4, and BIN1 With Pathological Diagnosis of Alzheimer Disease. JAMA Neurology. 2015 Jan 1;72(1):15–24.

87. Sobue A, Komine O, Hara Y, Endo F, Mizoguchi H, Watanabe S, et al. Microglial gene signature reveals loss of homeostatic microglia associated with neurodegeneration of Alzheimer’s disease. Acta Neuropathologica Communications. 2021 Jan 5;9(1):1.

88. Hsu CY, Lee KT, Sun TY, Sze CI, Huang SS, Hsu LJ, et al. WWOX and Its Binding Proteins in Neurodegeneration. Cells. 2021 Jul 14;10(7):1781.

89. Ullrich M, Bundschu K, Benz PM, Abesser M, Freudinger R, Fischer T, et al. Identification of SPRED2 (Sprouty-related Protein with EVH1 Domain 2) as a Negative Regulator of the Hypothalamic-Pituitary-Adrenal Axis. J Biol Chem. 2011 Mar 18;286(11):9477–88.

90. Styrpejko DJ, Cuajungco MP. Transmembrane 163 (TMEM163) Protein: A New Member of the Zinc Efflux Transporter Family. Biomedicines. 2021 Feb 21;9(2):220.

91. Bellenguez C, Küçükali F, Jansen IE, Kleineidam L, Moreno-Grau S, Amin N, et al. New insights into the genetic etiology of Alzheimer’s disease and related dementias. Nat Genet. 2022 Apr;54(4):412–36.

92. Mur J, McCartney DL, Chasman DI, Visscher PM, Muniz-Terrera G, Cox SR, et al. Variation in VKORC1 Is Associated with Vascular Dementia. J Alzheimers Dis. 80(3):1329–37.

93. Steiner J, Bogerts B, Schroeter ML, Bernstein HG. S100B protein in neurodegenerative disorders. Clin Chem Lab Med. 2011 Mar;49(3):409–24.

94. Mecocci P, Parnetti L, Romano G, Scarelli A, Chionne F, Cecchetti R, et al. Serum anti-GFAP and anti-S100 autoantibodies in brain aging, Alzheimer’s disease and vascular dementia. Journal of Neuroimmunology. 1995 Mar 1;57(1):165–70.

95. Tian J, Du E, Guo L. Mitochondrial Interaction with Serotonin in Neurobiology and Its Implication in Alzheimer’s Disease. J Alzheimers Dis Rep. 7(1):1165–77.

96. Davydova TV, Mikovskaya OI, Fomina VG, Voskresenskaya NI, Doronina OA. Induction of Immune Complexes and Autoantibodies to Serotonin and Dopamine in Patients with Alzheimer’s Disease. Bulletin of Experimental Biology and Medicine. 2002 Jul 1;134(1):23–5.

97. Zhou ZD, Yi LX, Wang DQ, Lim TM, Tan EK. Role of dopamine in the pathophysiology of Parkinson’s disease. Transl Neurodegener. 2023 Sep 18;12:44.

98. Lau A, Tymianski M. Glutamate receptors, neurotoxicity and neurodegeneration. Pflugers Arch. 2010 Jul;460(2):525–42.

99. Davydova TV, Voskresenskaya NI, Fomina VG, Vetrile LA, Doronina OA. Induction of autoantibodies to glutamate in patients with Alzheimer’s disease. Bull Exp Biol Med. 2007 Feb 1;143(2):182–3.

100. Dubey J, Ratnakaran N, Koushika SP. Neurodegeneration and microtubule dynamics: death by a thousand cuts. Front Cell Neurosci. 2015 Sep 9;9:343.

101. Terryberry JW, Thor G, Peter JB. Autoantibodies in Neurodegenerative Diseases: Antigen-Specific Frequencies and Intrathecal Analysis. Neurobiol Aging. 1998 May 1;19(3):205–16.

102. Abdelhak A, Foschi M, Abu-Rumeileh S, Yue JK, D’Anna L, Huss A, et al. Blood GFAP as an emerging biomarker in brain and spinal cord disorders. Nat Rev Neurol. 2022 Mar;18(3):158–72.

103. Tanaka J, Nakamura K, Takeda M, Tada K, Suzuki H, Morita H, et al. Enzyme-linked immunosorbent assay for human autoantibody to glial fibrillary acidic protein: higher titer of the antibody is detected in serum of patients with Alzheimer’s disease. Acta Neurologica Scandinavica. 1989;80(6):554–60.

104. Staabs F, Foverskov Rasmussen H, Buthut M, Höltje M, Li LY, Stöcker W, et al. Brain-targeting autoantibodies in patients with dementia. Front Neurol. 2024 Jul 10;15:1412813.

105. Wang K, Lu Y, Morrow DF, Xiao D, Xu C, Alzheimer’s Disease Neuroimaging Initiative. Associations of ARHGAP26 Polymorphisms with Alzheimer’s Disease and Cardiovascular Disease. J Mol Neurosci. 2022 May;72(5):1085–97.

106. Xie C, Kessi M, Yin F, Peng J. Roles of KCNA2 in Neurological Diseases: from Physiology to Pathology. Mol Neurobiol. 2024 Nov;61(11):8491–517.

107. Joubert B. The neurobiology and immunology of CASPR2-associated neurological disorders. Revue Neurologique. 2024 Nov 1;180(9):950–6.

108. Schaefer N, Roemer V, Janzen D, Villmann C. Impaired Glycine Receptor Trafficking in Neurological Diseases. Front Mol Neurosci. 2018 Aug 21;11:291.

109. Kim J, Lee S, Kang S, Kim SH, Kim JC, Yang M, et al. Brain-derived neurotropic factor and GABAergic transmission in neurodegeneration and neuroregeneration. Neural Regen Res. 2017 Oct;12(10):1733–41.

110. Gordon BA. Neurofilaments in disease: what do we know? Curr Opin Neurobiol. 2020 Apr;61:105–15.

111. Bartos A, Fialová L, Švarcová J, Ripova D. Patients with Alzheimer disease have elevated intrathecal synthesis of antibodies against tau protein and heavy neurofilament. J Neuroimmunol. 2012 Nov 15;252(1):100–5.

112. Imbimbo BP, Ippati S, Watling M, Imbimbo C. Role of monomeric amyloid-β in cognitive performance in Alzheimer’s disease: Insights from clinical trials with secretase inhibitors and monoclonal antibodies. Pharmacological Research. 2023 Jan 1;187:106631.

113. Wang YR, Zeng XQ, Wang J, Fowler CJ, Li QX, Bu XL, et al. Autoantibodies to BACE1 promote Aβ accumulation and neurodegeneration in Alzheimer’s disease. Acta Neuropathol. 2024 Oct 24;148(1):57.

114. Pasternak SH, Bagshaw RD, Guiral M, Zhang S, Ackerley CA, Pak BJ, et al. Presenilin-1, Nicastrin, Amyloid Precursor Protein, and γ-Secretase Activity Are Co-localized in the Lysosomal Membrane *. Journal of Biological Chemistry. 2003 Jul 18;278(29):26687–94.

115. Wang YR, Wang MT, Zeng XQ, Liu YH, Wang YJ, Yu JT. Associations of Naturally Occurring Antibodies to Presenilin-1 with Brain Amyloid-β Load and Cognitive Impairment in Alzheimer’s Disease. Journal of Alzheimer’s Disease. 2022 Jan 1;90(4):1493–500.

116. Biswas SC, Shi Y, Vonsattel JPG, Leung CL, Troy CM, Greene LA. Bim Is Elevated in Alzheimer’s Disease Neurons and Is Required for β-Amyloid-Induced Neuronal Apoptosis. J Neurosci. 2007 Jan 24;27(4):893–900.

117. Jian JM, Fan DY, Tian DY, Cheng Y, Sun PY, Tan CR, et al. Naturally-Occurring Antibodies Against Bim are Decreased in Alzheimer’s Disease and Attenuate AD-type Pathology in a Mouse Model. Neurosci Bull. 2022 Sep 1;38(9):1025–40.

118. Buck SA, Erickson-Oberg MQ, Bhatte SH, McKellar CD, Ramanathan VP, Rubin SA, et al. Roles of VGLUT2 and Dopamine/Glutamate Co-Transmission in Selective Vulnerability to Dopamine Neurodegeneration. ACS Chem Neurosci. 2022 Jan 19;13(2):187–93.

119. Hansen N, Teegen B, Hirschel S, Wiltfang J, Schott BH, Bartels C, et al. Case report: Mixed dementia associated with autoantibodies targeting the vesicular glutamate transporter 2. Front Psychiatry. 2023;14:1227824.

120. Morales-Briceño H, Cruse B, Fois AF, Lin MW, Jiang J, Banerjee D, et al. IgLON5-mediated neurodegeneration is a differential diagnosis of CNS Whipple disease. Neurology. 2018 Jun 12;90(24):1113–5.

121. Bastiaansen AEM, van Steenhoven RW, te Vaarwerk ES, van der Flier WM, Teunissen C, de Graaff E, et al. Antibodies Associated With Autoimmune Encephalitis in Patients With Presumed Neurodegenerative Dementia. Neurol Neuroimmunol Neuroinflamm. 2023 Jun 13;10(5):e200137.

122. Fels E, Muñiz-Castrillo S, Vogrig A, Joubert B, Honnorat J, Pascual O. Role of LGI1 protein in synaptic transmission: From physiology to pathology. Neurobiology of Disease. 2021 Dec 1;160:105537.

123. Wang R, Reddy PH. Role of glutamate and NMDA receptors in Alzheimer’s disease. J Alzheimers Dis. 2017;57(4):1041–8.

124. Busse M, Kunschmann R, Dobrowolny H, Hoffmann J, Bogerts B, Steiner J, et al. Dysfunction of the blood-cerebrospinal fluid-barrier and N-methyl-d-aspartate glutamate receptor antibodies in dementias. Eur Arch Psychiatry Clin Neurosci. 2018 Aug 1;268(5):483–92.

125. Busse S, Brix B, Kunschmann R, Bogerts B, Stoecker W, Busse M. N-methyl-d-aspartate glutamate receptor (NMDA-R) antibodies in mild cognitive impairment and dementias. Neurosci Res. 2014 Aug;85:58–64.

126. Jourdi G, Fleury S, Boukhatem I, Lordkipanidzé M. Soluble p75 neurotrophic receptor as a reliable biomarker in neurodegenerative diseases: what is the evidence? Neural Regeneration Research. 2024 Mar;19(3):536.

127. He CY, Tian DY, Chen SH, Jin WS, Cheng Y, Xin JY, et al. Elevated Levels of Naturally-Occurring Autoantibodies Against the Extracellular Domain of p75NTR Aggravate the Pathology of Alzheimer’s Disease. Neurosci Bull. 2023 Feb;39(2):261–72.

128. Shelly S, Kryzer TJ, Komorowski L, Miske R, Anderson MD, Flanagan EP, et al. Neurochondrin neurological autoimmunity. Neurol Neuroimmunol Neuroinflamm. 2019 Nov;6(6):e612.

129. Hansen N, Malchow B, Teegen B, Wiltfang J, Bartels C. Case Report: Alzheimer’s Dementia Associated With Cerebrospinal Fluid Neurochondrin Autoantibodies. Front Neurol. 2022;13:879009.

130. San Segundo-Acosta P, Montero-Calle A, Jernbom-Falk A, Alonso-Navarro M, Pin E, Andersson E, et al. Multiomics Profiling of Alzheimer’s Disease Serum for the Identification of Autoantibody Biomarkers. J Proteome Res. 2021 Oct 10;

131. Biembengut ÍV, Silva ILZ, Souza T de ACB de, Shigunov P. Cytoplasmic FMR1 interacting protein (CYFIP) family members and their function in neural development and disorders. Mol Biol Rep. 2021 Aug 1;48(8):6131–43.

132. Costessi L, Porro F, Iaconcig A, Muro AF. TDP-43 regulates β-adducin (Add2) transcript stability. RNA Biol. 2014;11(10):1280–90.

133. Barrick BJ, Ida CM, Laniosz V, Jentoft ME, Sominidi-Damodaran S, Wieland CN, et al. Bullous Pemphigoid, Neurodegenerative Disease, and Hippocampal BP180 Expression: A Retrospective Postmortem Neuropathologic Study. J Invest Dermatol. 2016 Oct 1;136(10):2090–2.

134. Wang YN, Hammers CM, Mao X, Jin HZ, Yuan J, Li L. Analysis of the autoimmune response against BP180 in patients with Alzheimer’s disease. Ann Transl Med. 2021 Jan;9(2):107.

135. Kokkonen N, Herukka SK, Huilaja L, Kokki M, Koivisto AM, Hartikainen P, et al. Increased Levels of the Bullous Pemphigoid BP180 Autoantibody Are Associated with More Severe Dementia in Alzheimer’s Disease. J Invest Dermatol. 2017 Jan 1;137(1):71–6.

136. Tuusa J, Lindgren O, Tertsunen HM, Nishie W, Kokkonen N, Huilaja L, et al. BP180 Autoantibodies Target Different Epitopes in Multiple Sclerosis or Alzheimer’s Disease than in Bullous Pemphigoid. J Invest Dermatol. 2019 Feb;139(2):293–9.

137. Perez DM. α1-Adrenergic Receptors: Insights into Potential Therapeutic Opportunities for COVID-19, Heart Failure, and Alzheimer’s Disease. Int J Mol Sci. 2023 Feb 20;24(4):4188.

138. Hempel P, Heinig B, Jerosch C, Decius I, Karczewski P, Kassner U, et al. Immunoadsorption of Agonistic Autoantibodies Against α1-Adrenergic Receptors in Patients With Mild to Moderate Dementia. Ther Apher Dial. 2016 Oct;20(5):523–9.

139. Karczewski P, Hempel P, Kunze R, Bimmler M. Agonistic autoantibodies to the α(1) -adrenergic receptor and the β(2) -adrenergic receptor in Alzheimer’s and vascular dementia. Scand J Immunol. 2012 May;75(5):524–30.

140. Torrente D, Su EJ, Schielke GP, Warnock M, Mann K, Lawrence DA. Opposing effects of β-2 and β-1 adrenergic receptor signaling on neuroinflammation and dopaminergic neuron survival in α-synuclein-mediated neurotoxicity. Journal of Neuroinflammation. 2023 Mar 2;20(1):56.

141. Reindl M, Waters P. Myelin oligodendrocyte glycoprotein antibodies in neurological disease. Nat Rev Neurol. 2019 Feb;15(2):89–102.

142. Papuć E, Kurys-Denis E, Krupski W, Tatara M, Rejdak K. Can Antibodies Against Glial Derived Antigens be Early Biomarkers of Hippocampal Demyelination and Memory Loss in Alzheimer’s Disease? J Alzheimers Dis. 2015;48(1):115–21.

143. Mukhopadhyay G, Doherty P, Walsh FS, Crocker PR, Filbin MT. A novel role for myelin-associated glycoprotein as an inhibitor of axonal regeneration. Neuron. 1994 Sep;13(3):757–67.

144. Garbern JY, Yool DA, Moore GJ, Wilds IB, Faulk MW, Klugmann M, et al. Patients lacking the major CNS myelin protein, proteolipid protein 1, develop length-dependent axonal degeneration in the absence of demyelination and inflammation. Brain. 2002 Mar;125(Pt 3):551–61.

145. Frid K, Einstein O, Friedman-Levi Y, Binyamin O, Ben-Hur T, Gabizon R. Aggregation of MBP in chronic demyelination. Ann Clin Transl Neurol. 2015 Jul;2(7):711–21.

146. Singh VK, Yang YY, Singh EA. Immunoblot detection of antibodies to myelin basic protein in Alzheimer’s disease patients. Neurosci Lett. 1992 Nov 23;147(1):25–8.

147. Weng PH, Chen JH, Chen TF, Sun Y, Wen LL, Yip PK, et al. CHRNA7 Polymorphisms and Dementia Risk: Interactions with Apolipoprotein ε4 and Cigarette Smoking. Sci Rep. 2016 Jun 2;6(1):27231.

148. Kamynina AV, Ponomareva EV, Koroev DO, Volkova TD, Kolykhalov IV, Selezneva ND, et al. [The reduced level of antibodies to acetylcholine receptor alpha 7 fragment in the blood serum of patients with Alzheimer’s disease]. Zh Nevrol Psikhiatr Im S S Korsakova. 2015;115(12):128–32.

149. Sipione S, Monyror J, Galleguillos D, Steinberg N, Kadam V. Gangliosides in the Brain: Physiology, Pathophysiology and Therapeutic Applications. Front Neurosci [Internet]. 2020 Oct 6 [cited 2024 Dec 20];14. Available from: https://www.frontiersin.org/journals/neuroscience/articles/10.3389/fnins.2020.572965/full

150. Hatzifilippou E, Koutsouraki E, Costa VG, Baloyannis SJ. Antibodies against gangliosides in patients with dementia. Am J Alzheimers Dis Other Demen. 2014 Dec;29(8):660–6.

151. Hatzifilippou E, Koutsouraki E, Banaki T, Traka M, Costa VG, Baloyannis SJ. Antibodies against GM1 in demented patients. Am J Alzheimers Dis Other Demen. 2008 Jul;23(3):274–9.

152. Ariga T, Kubota M, Nakane M, Oguro K, Yu RK, Ando S. Anti-Chol-1 antigen, GQ1bα, antibodies are associated with Alzheimer’s disease. PLoS One. 2013;8(5):e63326.

153. Chapman J, Sela BA, Wertman E, Michaelson DM. Antibodies to ganglioside GM1 in patients with Alzheimer’s disease. Neurosci Lett. 1988 Mar 31;86(2):235–40.

154. Bennett MC. The role of alpha-synuclein in neurodegenerative diseases. Pharmacol Ther. 2005 Mar;105(3):311–31.

155. Koehler NKU, Stransky E, Shing M, Gaertner S, Meyer M, Schreitmüller B, et al. Altered serum IgG levels to α-synuclein in dementia with Lewy bodies and Alzheimer’s disease. PLoS One. 2013;8(5):e64649.

156. Counts SE, Perez SE, Ginsberg SD, Mufson EJ. Neuroprotective Role for Galanin in Alzheimer’s Disease. EXS. 2010;102:143–62.

157. Costa A, Bini P, Hamze-Sinno M, Moglia A, Franciotta D, Sinforiani E, et al. Galanin and α-MSH autoantibodies in cerebrospinal fluid of patients with Alzheimer’s disease. J Neuroimmunol. 2011 Dec 15;240–241:114–20.

158. Peng T, Wang J, Lu J, Lu H, Teng J, Jia Y. Neuroprotective effects of α-melanocyte-stimulating hormone against the neurotoxicity of 1-methyl-4-phenylpyridinium. IUBMB Life. 2017;69(5):315–20.

159. Wang J, Roy SK, Xu Y. Spatiotemporal expression and coexpression patterns of SRPK1 in the human brain: A neurodevelopmental perspective. Brain Behav. 2023 Dec 31;14(1):e3341.

160. Daniilidou M, Tsolaki M, Giannakouros T, Nikolakaki E. Detection of elevated antibodies against SR protein kinase 1 in the serum of Alzheimer’s disease patients. J Neuroimmunol. 2011 Sep 15;238(1):67–72.

161. Ebanks B, Chakrabarti L. Mitochondrial ATP Synthase is a Target of Oxidative Stress in Neurodegenerative Diseases. Front Mol Biosci [Internet]. 2022 Feb 14 [cited 2024 Dec 20];9. Available from: https://www.frontiersin.org/journals/molecular-biosciences/articles/10.3389/fmolb.2022.854321/full

162. Vacirca D, Delunardo F, Matarrese P, Colasanti T, Margutti P, Siracusano A, et al. Autoantibodies to the adenosine triphosphate synthase play a pathogenetic role in Alzheimer’s disease. Neurobiology of Aging. 2012 Apr 1;33(4):753–66.

163. Walter A, Korth U, Hilgert M, Hartmann J, Weichel O, Hilgert M, et al. Glycerophosphocholine is elevated in cerebrospinal fluid of Alzheimer patients. Neurobiol Aging. 2004;25(10):1299–303.

164. Eriksson UK, Sjöberg BG, Bennet AM, de Faire U, Pedersen NL, Frostegård J. Low levels of antibodies against phosphorylcholine in Alzheimer’s disease. J Alzheimers Dis. 2010;21(2):577–84.

165. Satoh K, Kawakami A, Shirabe S, Tamai M, Sato A, Tsujihata M, et al. Anti-cyclic citrullinated peptide antibody (anti-CCP antibody) is present in the sera of patients with dementia of Alzheimer’s type in Asian. Acta Neurol Scand. 2010 May;121(5):338–41.

166. Sugawa M, Ikeda S, Kushima Y, Takashima Y, Cynshi O. Oxidized low density lipoprotein caused CNS neuron cell death. Brain Research. 1997 Jun 27;761(1):165–72.

167. Kankaanpää J, Turunen SP, Moilanen V, Hörkkö S, Remes AM. Cerebrospinal fluid antibodies to oxidized LDL are increased in Alzheimer’s disease. Neurobiol Dis. 2009 Mar;33(3):467–72.

168. Guo T, Zhou L, Xiong M, Xiong J, Huang J, Li Y, et al. N-homocysteinylation of DJ-1 promotes neurodegeneration in Parkinson’s disease. Aging Cell. 2024;23(5):e14124.

169. Włoczkowska O, Perła‐Kaján J, Smith AD, de Jager C, Refsum H, Jakubowski H. Anti‐N‐homocysteine‐protein autoantibodies are associated with impaired cognition. Alzheimers Dement (N Y). 2021 Mar 31;7(1):e12159.

170. Xu X, Huang E, Tai Y, Zhao X, Chen X, Chen C, et al. Nupr1 Modulates Methamphetamine-Induced Dopaminergic Neuronal Apoptosis and Autophagy through CHOP-Trib3-Mediated Endoplasmic Reticulum Stress Signaling Pathway. Front Mol Neurosci. 2017 Jun 26;10:203.

171. San Segundo-Acosta P, Montero-Calle A, Fuentes M, Rábano A, Villalba M, Barderas R. Identification of Alzheimer’s Disease Autoantibodies and Their Target Biomarkers by Phage Microarrays. J Proteome Res. 2019 Jul 5;18(7):2940–53.

172. Yang C, Wang H, Shao M, Chu F, He Y, Chen X, et al. Brain-Type Glycogen Phosphorylase (PYGB) in the Pathologies of Diseases: A Systematic Review. Cells. 2024 Feb 5;13(3):289.
